# Global DNA Hypermethylation in Down Syndrome Placenta

**DOI:** 10.1371/journal.pgen.1003515

**Published:** 2013-06-06

**Authors:** Shengnan Jin, Yew Kok Lee, Yen Ching Lim, Zejun Zheng, Xueqin Michelle Lin, Desmond P. Y. Ng, Joanna D. Holbrook, Hai Yang Law, Kenneth Y. C. Kwek, George S. H. Yeo, Chunming Ding

**Affiliations:** 1 Growth, Development and Metabolism Program, Singapore Institute for Clinical Sciences, Agency for Science, Technology and Research, Singapore; 2 KK Women's and Children's Hospital, Singapore; Centre for Cancer Biology, SA Pathology, Australia

## Abstract

Down syndrome (DS), commonly caused by an extra copy of chromosome 21 (chr21), occurs in approximately one out of 700 live births. Precisely how an extra chr21 causes over 80 clinically defined phenotypes is not yet clear. Reduced representation bisulfite sequencing (RRBS) analysis at single base resolution revealed DNA hypermethylation in all autosomes in DS samples. We hypothesize that such global hypermethylation may be mediated by down-regulation of TET family genes involved in DNA demethylation, and down-regulation of *REST/NRSF* involved in transcriptional and epigenetic regulation. Genes located on chr21 were up-regulated by an average of 53% in DS compared to normal villi, while genes with promoter hypermethylation were modestly down-regulated. DNA methylation perturbation was conserved in DS placenta villi and in adult DS peripheral blood leukocytes, and enriched for genes known to be causally associated with DS phenotypes. Our data suggest that global epigenetic changes may occur early in development and contribute to DS phenotypes.

## Author Summary

Down syndrome (DS) occurs in approximately one out of 700 live births. DS is caused by an extra copy of chromosome 21. Although over 80 clinically defined phenotypes are identified for DS, each affected individual may only show some of the disease phenotypes. Understanding how the extra chromosome 21 causes various disease phenotypes can lead to better management and over the long term, treatment of the individuals with DS to improve outcome. In this study, we looked into DNA methylation changes associated with DS placenta villi tissues. We found genes with perturbed DNA methylation in promoters are functionally relevant to DS phenotypes. Through gene expression analysis, we identified genes (*TET1, TET2, REST*) that may contribute to the perturbed DNA methylation in DS.

## Introduction

Genomic copy variations ranging from copy number variations to chromosome aneuploidies offer biological diversity and are also a common cause of genetic disorders. Down syndrome (DS), caused by triplication of chromosome 21 (chr21), is characterized by over 80 clinically defined phenotypes of different penetrance and expressivity affecting many different organs such as the central nervous system, heart, gastrointestinal tract, and immune system [1]. Since the genetic basis for DS is clearly caused by an extra copy (occasionally a partial extra copy) of chr21, many studies focused on genes located on chr21. Many, but clearly not all, genes located on chr21 are expressed at higher levels in individuals with DS or mouse models [2]–[4]. Meanwhile, many genes on other chromosomes were also dys-regulated [5]–[7]. How an extra chr21 causes global gene expression dys-regulation and how such dys-regulation contributes to DS phenotypes remain to be addressed.

Epigenetic regulation of gene expression is one important mechanism in development and disease. In the nervous system, many key enzymes such as *DNMT1*, *DNMT3A*, and *TET1* for epigenetic regulation are abundantly expressed [8], [9]. Epigenetic alternations are frequently observed in intellectual disability syndromes [10]. For example, Rett syndrome may be caused by mutations in *MECP2* [11]. In psychosis, DNA hypermethylation was observed, presumably due to elevated levels of methyl donor S-adenosylmethionine (SAM), and *DNMT1* over-expression [12].

In DS, genes such as *DYRK1A* located on chr21 are potential candidates causing disorders in the nervous system [13]. Homocysteine metabolism is perturbed in children with DS, resulting in lower levels of SAM and S-adenosylhomocysteine (SAH) [14]. Small-scale DNA methylation analyses were performed to study potential DNA methylation perturbations in DS [15]–[18]. Intriguingly, promoter hypermethylation was observed in DS [18], despite of lower levels of SAM.

To understand, at epigenome level, the potential perturbations associated with DS, and whether such perturbations are functionally relevant to DS, we quantified CpG methylation at single base resolution in 17 placenta villi samples (11 DS and six normal samples) with an improved version of reduced representation bisulfite sequencing (RRBS). We further quantified the transcriptome in placenta villi (four DS and five normal samples). A global hypermethylation in all genomic regions and all autosomes were observed in DS samples, with genes with promoter hypermethylation enriched for functions relevant to DS phenotypes. Our data suggest epigenetic perturbation may be one important mechanism linking the most common chromosomal aneuploidy and its phenotypes.

## Results

**Figure 1:**
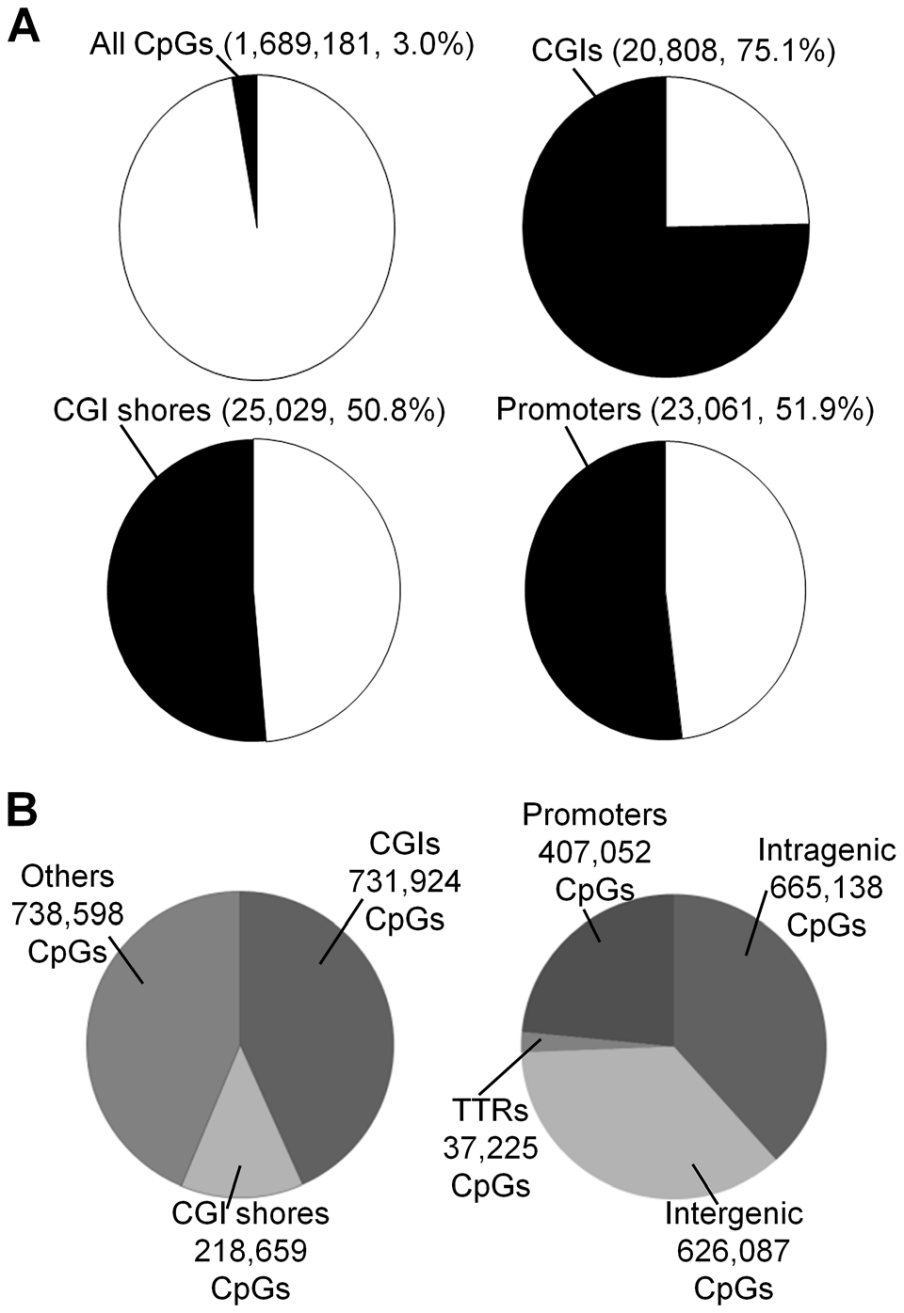
Coverage of CpGs for RRBS analysis. (**A**) A CpG site was considered covered if the sequencing depth was ≥10. A genomic region (CGI, CGI shore or promoter) was considered covered if at least 3 CpGs within the region was sequenced at a depth ≥10. (**B**) Distributions of covered CpGs in different functional regions. CGIs: CpG islands.

RRBS was used to quantify DNA methylation. On average, about 1.7 million CpG sites with a sequencing depth ≥10 (minimum sequencing depth of 10 is used in all subsequent analyses, unless specified otherwise) in each of 17 placenta villi samples (11 DS and six normal samples) ( Table S1 and Figure S1A-S1B). Principal component analysis revealed separation of samples based on disease status (normal or DS), but not on gender (Figure S1C). Assayed CpG sites represent about 3.0% of all CpG sites in the human genome (on both the forward and the reverse strands) (Figure 1A), spreading across regions that are CpG rich (CpG islands, 731,924 CpGs), CpG medium rich (CpG island shores, defined as 2-kb upstream or downstream of CGIs, 218,659 CpGs), and other genomic regions (738,598 CpGs) (Figure 1B). The covered CpGs were distributed in promoters (defined as −1000 bp to +500 relative to a transcription start site, 407,052 CpGs), intragenic regions (665,138 CpGs), intergenic regions (626,087 CpGs) and transcription termination regions (TTRs, defined as −500 to +500 relative to a transcription termination site, 37,225 CpGs) (Figure 1B). On average, 20,808 CGIs, 25,029 CGI shores and 23,061 promoters (Figure 1A) were covered for each individual sample, representing 75.1%, 50.8% and 51.9% of all such regions in the human genome, respectively [19].

Two technical replicates for one sample (sample T3 in Table S1) with independent bisulfite conversions were reproducible (r = 0.957, Figure S2A). We also compared our data with a published report using Illumina HumanMethylation27K BeadChip [18] and identified 2,894 CpGs that were analyzed by both data sets. Good correlation was observed for both normal (r = 0.929) and DS samples (r = 0.913) (Figure S2B–S2C).

The methylation levels of the CpGs showed a bimodal distribution pattern with ∼30% of the CpGs at 0–5% methylation, and ∼10% of the CpGs at 95–100% methylation (Figure S3A), consistent with earlier large-scale DNA methylome studies in other cell types [20]–[25], although the proportion of fully methylated CpGs was substantially lower in this study due to the intentional RRBS design to remove repetitive sequences. The distributions of methylation levels for CpGs from different functional locations (promoters, TTRs, intragenic, and intergenic regions) were dramatically different (Figure S3B–S3E). CpGs in the promoters were much more enriched in the 0–5% methylation level while very few CpGs were methylated at levels higher than 20%. Higher proportions of CpG sites were partially methylated (30–70% methylation level) in non-promoter regions, an observation also made by others [24].

We next assessed the inter-individual variability in CpG methylation [26] in the five normal samples with male fetuses. We selected partially methylated CpGs (average methylation 30–70% in the five samples) since these CpGs were likely to be most variable. At a minimum sequencing depth cut-off of 10, 20, or 50, the overall variability levels measured by standard deviations in the five samples for each partially methylated CpGs were relatively low (Figure S4), typically below 10%. Interestingly, a number of CpGs were highly variable among the five normal samples.

**Figure 2:**
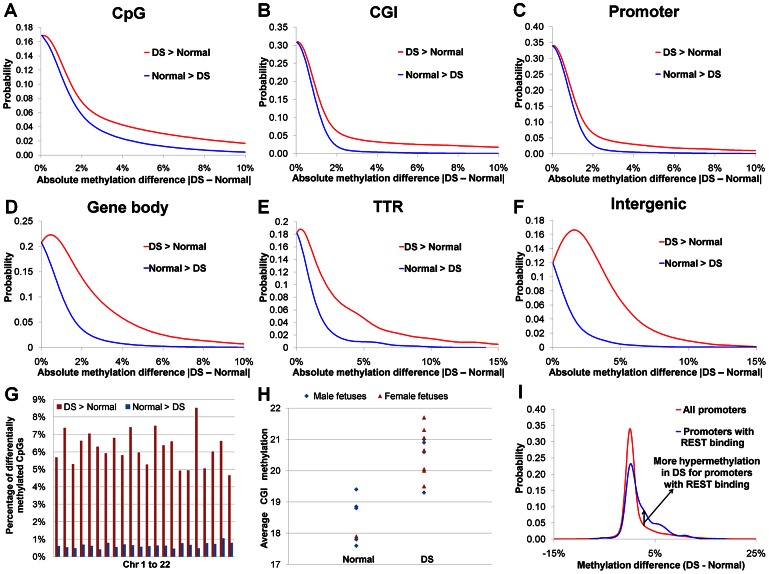
DNA methylation perturbations in DS. (**A**) Probability density function (PDF) distribution for methylation difference between DS and normal samples for individual CpGs. Similarly, the methylation difference values for (**B**) 18,939 CGIs (each CGI with at least 6 covered CpGs), (**C**) 19,479 promoters (each promoter with at least 6 covered CpGs), (**D**) 30,648 gene bodies (each gene body with at least 6 covered CpGs), (**E**) 3,215 TTRs (each TTR with at least 6 covered CpGs) and (**F**) 8,611 intergenic regions (each intergenic region with at least 6 covered CpGs) were used for calculating their respective PDF distributions. In (**A–F**), hypermethylation in DS (DS>Normal) occurs much more frequently than hypomethylation in DS (Normal>DS). (**G**) Percentages of hyper- and hypomethylated CpGs in each autosome. (**H**) Average CGI methylation was higher in DS than in normal samples (*p*<0.002, Wilcoxon rank-sum test, two-sided). Only CGIs with at least 6 covered CpGs were included. (**I**) PDF distributions of methylation difference for all promoters and promoters targeted by REST.

We observed a global DNA hypermethylation in DS samples. Earlier reports showed that hypermethylated promoters outnumbered hypomethylated promoters in DS chorionic villus samples and leukocytes [18], [27]. In our study, dominance of hypermethylation over hypomethylation in DS was seen in all genomic regions (promoters, intragenic regions, intergenic regions and transcription termination regions, Figure 2A–2F, Table S2), and in all autosomes (Figure 2G). Such dominance of hypermethylation was most pronounced in promoter regions, particularly promoters overlapping with CGIs (hypermethylated CpG number/hypomethylated CpG number: 56.2). The average CGI methylation levels in individual DS samples were also higher than those of normal samples (*p*<0.002, Wilcoxon rank-sum test, two-sided) (Figure 2H). Global hypermethylation in DS (not limited or even enriched in chr21) is different from X chromosome-specific DNA hypermethylation in females as hypermethylation in the latter is largely confined to the X chromosome.

Studies on differential DNA methylation have traditionally been focused on CGIs and promoters. In our study, differential DNA methylation (hypermethylated and hypomethylated CpGs) was most frequent in intergenic and TTR regions, followed closely by intragenic regions. Promoters, particularly those overlapping with CGIs, were the least likely to be differentially methylated due to DS (Table S2), consistent with recent genome-wide DNA methylation studies [24], [25].

Differential DNA methylation in DS showed conservation in different tissues and across the life course. Out of the nine genes with differential DNA methylation between peripheral blood leukocytes (PBLs) from DS adults and karyotypically normal controls reported by Kerkel *et al.* [27], three genes (*TCF7*, *FAM62C*, and *CPT1B*) were also similarly differentially methylated in the placenta villi in this study (*p*<1.8×10^−9^, see methods). Differential DNA methylation of these genes was further validated by the EpiTYPER assays using gestational age matched samples (14 normal and 17 DS samples, Table S3, Figure S5A–S5B). The placenta is of extraembryonic origin while the PBLs are derived from the embryo proper. Significant conservation in DNA methylation perturbation in these two samples of different developmental origins suggests that DNA methylation perturbation in DS may occur very early in development.

**Figure 3:**
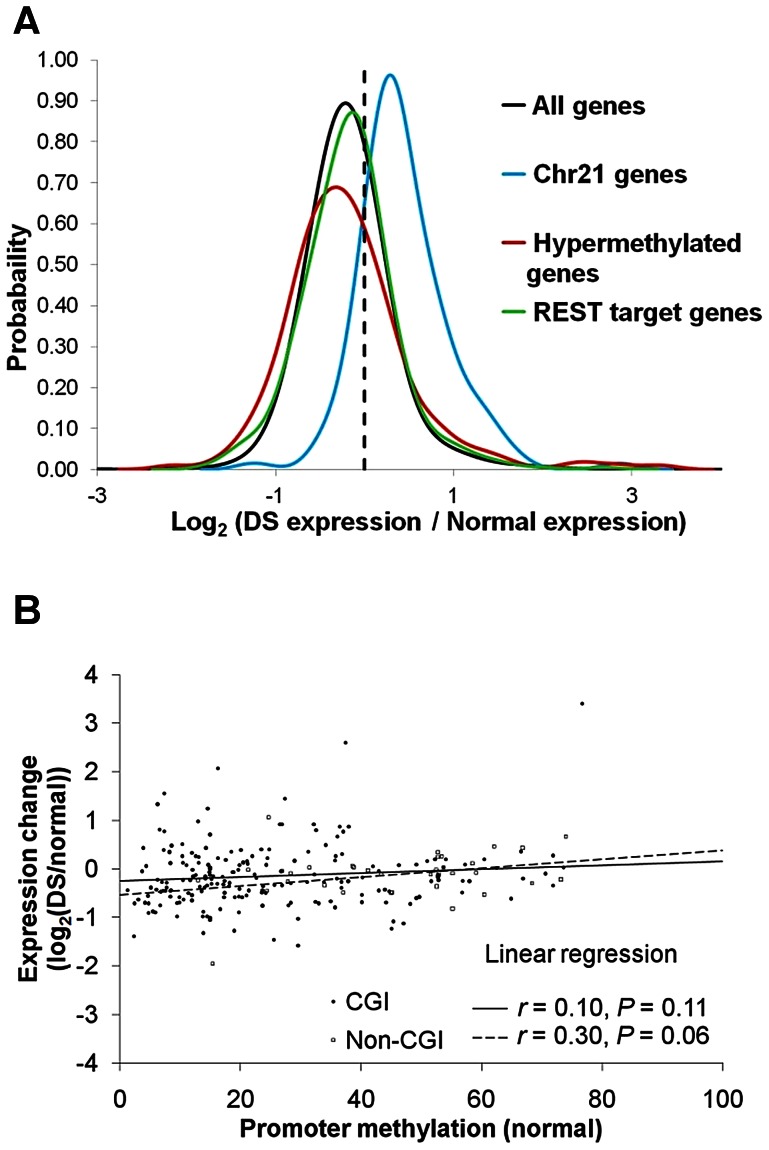
Gene expression changes in DS. For each gene, average expression values (RPKM) were calculated for both normal and DS samples. Only genes with RPKM ≥0.5 in at least one sample group were used for further analysis. Gene expression changes in DS were represented by log2 (Average DS samples expression/Average normal samples expression). (**A**) PDF distribution for gene expression changes for all genes, chr21 genes, hypermethylated genes and REST target genes. Genes located on chr21 were up-regulated by an average of 53% in DS. The hypermethylated genes were down-regulated, as evidenced by a left shift of the PDF curve (*p*<0.05, Wilcoxon rank-sum test, two-sided). The genes targeted by REST were marginally up-regulated (*p* = 0.06, Wilcoxon rank-sum test, two-sided). (**B**) Repression of gene expression by promoter hypermethylation was more prominent in promoters that were originally at lower methylation levels in normal samples. Each data point represents one hypermethylated promoter. X axis is the average methylation level in the normal samples for each promoter. Y axis is the gene expression ratio between DS and normal (log2 transformed).

We next performed RNA-Seq analysis in five normal and four DS placenta villi samples (Table S1). Genes located on chr21 were up-regulated by an average of 53% in DS (Figure 3A), consistent with previous reports [2]–[4]. _ENREF_10_ENREF_10Many well-studied genes such as *BACH1*, *SOD1*, *TIAM1*, *ITSN1*, *DSCR1/RCAN1*, and *DYRK1A* located on chr21 were up-regulated (Table S4). A total of 589 genes across all autosomes were hypermethylated in the promoters in DS. Out of the 589 genes, 207 genes passed the expression threshold (reads per kilobase per million mapped reads, RPKM≥0.5) and are located on autosomes other than chr21. Significant down-regulation of gene expression was observed for the 207 genes (*p*<0.05, Wilcoxon rank-sum test, two-sided). Interestingly, the association between promoter hypermethylation and gene expression repression was more pronounced for promoters with lower DNA methylation in the normal samples, suggesting that increased methylation in originally unmethylated promoters is likely to have a bigger impact on gene expression (Figure 3B). We further validated four genes (*CES1*, *TFAP2E*, *CDH13*, *NDN*) that showed increased promoter methylation and decreased gene expression, with EpiTYPER assays and quantitative real-time PCR, with a new set of gestational age matched samples (Table S3, Figure S6A–S6B).

An overall DNA hypermethylation in DS is intriguing since reduced levels of SAM (a primary methyl donor) and SAH were observed in the plasma of individuals with DS [14], suggesting enzymes regulating DNA methylation, instead of the availability of methyl donor molecules, are involved.

To explore potential pathways leading to global DNA hypermethylation in DS, we investigated the expression changes for several groups of genes involved in epigenetic regulation (Table S4). The TET family genes (*TET1* (chr10), *TET2* (chr4), and *TET3* (chr2)) involved in DNA demethylation [9], [28]–[30] were all down-regulated in DS. *TET1* and *TET2* down-regulation was further validated with quantitative real-time PCR on a new set of gestational age matched samples (Table S3, Figure S7A–S7B), while *TET3* down-regulation was not statistically significant (Figure S7C). Global DNA hypermethylation was previously observed in *TET1* knockdown mouse ES cells [31]. *TET1*−/− mice were viable, with deficiency in adult neurogenesis (Cui Q.Y. *et al.*, manuscript under review) and smaller body size [32], phenotypes also observed in DS [33]–[37]. Notably, CpG hypermethylation in DS was indeed 50% more frequent in TET target regions enriched for 5′-hydroxylmethylcytosine [38].

We carried out pathway and process network analyses for 598 genes with differential methylation in their promoters (hypermethylation: 589, hypomethylation: 9) in DS with a commercial database (MetaCore from GeneGo Inc.). The three significantly enriched (Hypergeometric *p*<0.05, corrected for multiple testing) pathway maps were “Immune response_Lectin induced complement pathway [39]”, “neurophysiological process Dopamine D2 receptor signaling in CNS” and “cytoskeleton remodeling Neurofilaments” (Figure S8A–S8D). Each pathway contained five differentially methylated genes without overlapping genes among the pathways. The three significantly enriched process networks were “Inflammation Complement system”, “Signal transduction Neuropeptide signaling pathways”, “Developmental Neurogenesis Axonal guidance” (Figure S8E). Both analyses pointed to perturbations in the physiology and activity of the neurons, consistent with cognitive impairment and neuronal degeneration being the most prevalent DS phenotypes, and perturbations in the immune system.

In addition, eight of the 598 differentially methylated promoters were included in the GeneGO list with causal association to DS (Table S5). This represents a significant enrichment for DS causally associated genes (*p*<0.05, permutation test, 1000 permutations, assuming a universe of 15,203 background genes).

Genes targeted by repressor element 1 silencing transcription factor (*REST*), aka *NRSF*, were found to be enriched for differential promoter methylation in DS (Figure S9, Table S6). *REST* is a transcriptional and epigenetic regulator in both neuronal and non-neural cells (e.g. heart) [40]. Decreased *REST* mRNA levels were found in cultured fetal DS brain cell-derived neurospheres [41]. In the placental villi, we also found a down-regulation of *REST* gene expression in DS samples (Table S4) by RNA-Seq, and quantitative real-time PCR on a set of gestational age matched samples (Figure S7D, *p*<0.05, t-test, two-sided). Recent work by Stadler *et al.* demonstrated that REST binding to its target regions was sufficient and necessary to maintain DNA hypomethylation in what they called low-methylated regions [24]. In *REST* −/− cells DNA hypermethylation was observed [24]. Down-regulation of REST in DS may lead to reduced binding of REST to its target genes, resulting in DNA hypermethylation in the target regions (Figure 2I). REST target genes were marginally up-regulated (Figure 3A, *p* = 0.06, Wilcoxon rank-sum test, two-sided), consistent with REST being largely a repressor in gene expression.

## Discussion

**Figure 4:**
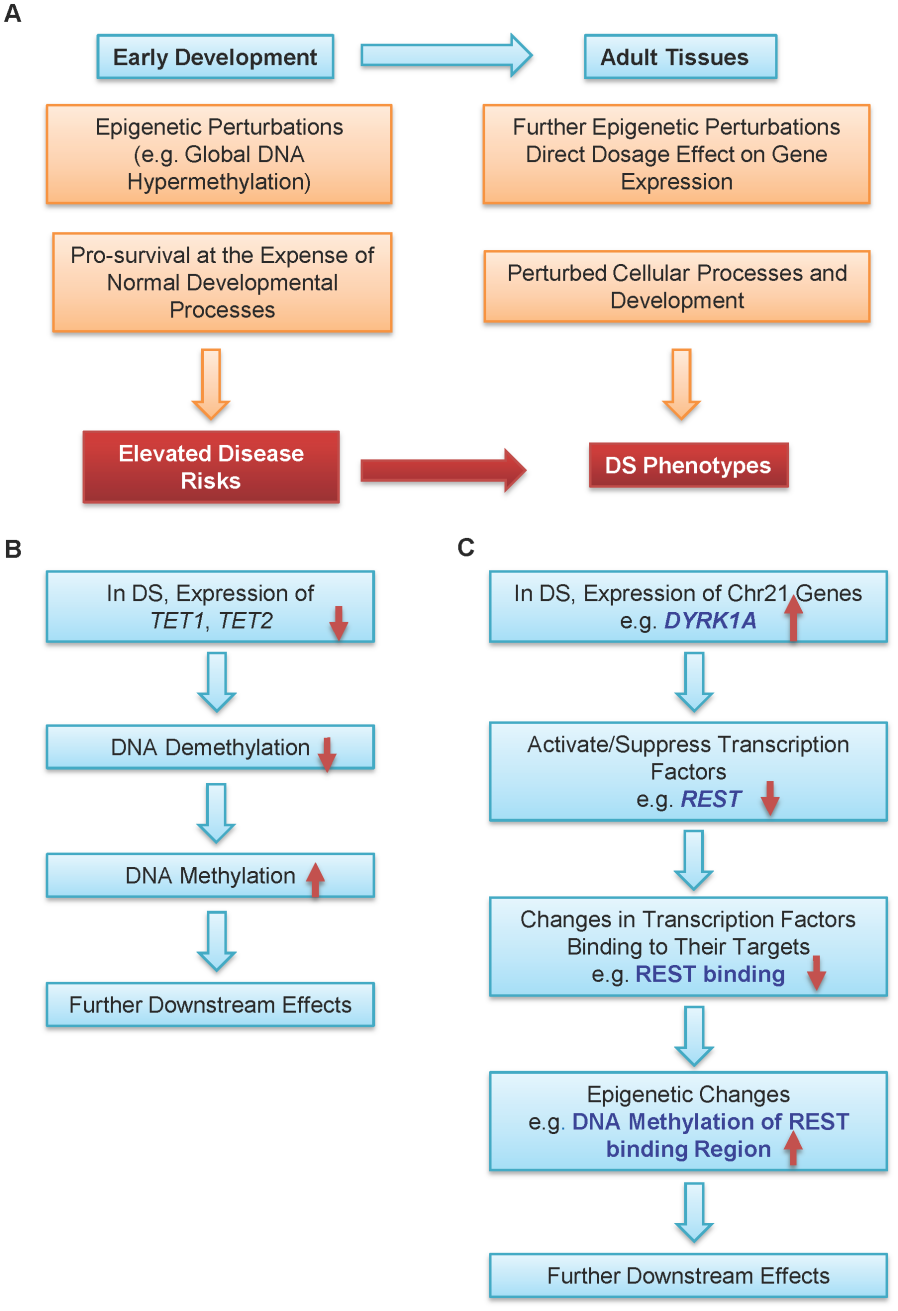
A model for epigenetic contributions to DS phenotypes. (**A**) Epigenetic perturbations such as global DNA methylation are early events in development. Consequently, multiple different adult tissues may share common patterns of epigenetic perturbations. Such early perturbations in response to an extra chr21 may confer certain survival advantages, at the expense of some normal developmental processes, which may lead to elevated disease risks. Further epigenetic perturbations and other abnormalities may alter cellular processes and development. Collectively, these perturbations may help contribution to many DS phenotypes with different penetrance and expressivity. Hypermethylation in DS may be caused by (**B**) down-regulation of the TET family genes and (**C**) down-regulation of *REST* by *DYRK1A* located on chr21.

We propose that epigenetic regulation is one possible mechanism connecting Trisomy 21 and DS phenotypes (Figure 4A). A persistent epigenetic perturbation may occur in DS embryos early in development, as supported by three out of the nine genes being similarly differentially methylated in the placenta villi in early gestation and peripheral blood leukocytes in adulthood. Such early perturbation may confer certain survival advantages, while leaving individuals with DS suffering from developmental defects and elevated risks to certain diseases. Additional epigenetic perturbations may occur later in development, further contributing to various DS phenotypes. Data from other groups and this study also provided two possible pathways leading to global DNA hypermethylation in DS. Down-regulation of the TET family genes may lead to hypermethylation of their target regions through decreased DNA demethylation (Figure 4B). Elevated expression of *DYRK1A*, a gene located in the DS critical region on chr21, may induce global epigenetic changes via down-regulating *REST* expression to cause hypermethylation of REST target genes (Figure 4C). *DYRK1A* mediates down-regulation of REST and interacts with the REST–SWI/SNF chromatin remodeling complex in mouse Trisomy 21 models [37], [42]. Global hypermethylation may also be mediated by other enzymes involved in epigenetic regulation of histone modifications.

Cautions should be taken for interpreting DNA methylome data derived from the placenta tissues as there are multiple confounding factors such as gestational age of the placenta [43], gender, and potentially different cell type mixtures from different samples. For both DNA methylation and gene expression, we validated a number of genes using a new set of gestational age matched samples (normal and DS), with EpiTYPER (for DNA methylation) and quantitative real-time PCR (for gene expression). We also excluded the X and Y chromosomes from differential DNA methylation analysis since the female X chromosome is known to be hypermethylated compared with the male X chromosome.

A few issues remain to be addressed in our model. First, how are the TET genes down-regulated in DS. To our knowledge, regulation of TET genes is not yet well understood. Are chr21 genes directly involved in the down-regulation, or is it an indirect effect? Segmental trisomies [44], [45] may be useful in mapping chr21 genes involved in TET genes regulation. Second, bisulfite sequencing does not distinguish between 5-hydroxylmethylcytosine (5hmC) and 5-methylcytosine (5mC). Is there a concurrent decrease in 5hmC level for the hypermethylated regions in DS? Third, the functional roles of the two pathways in our model need further characterization, possibly in cell lines or tissues relevant to specific DS phenotypes. Fourth, other potential pathways with epigenetic perturbations in DS remain to be further elucidated. It would be interesting to ask whether epigenetics plays a role for these genes to affect phenotypes. Additionally, it should be noted that although some epigenetic perturbations may be conserved in different tissues, the functional effects of epigenetic perturbations are likely to be temporal and spatial specific. To decipher the exact mechanisms for various DS phenotypes, studies on other tissues at different developmental stages may be necessary, possibly using murine models. Hopefully, a better understanding of the molecular and cellular abnormalities associated with DS may lead to new therapies for the sequela of DS, such as cognitive and developmental defects [46], [47].

## Materials and Methods

### Ethics statement

Informed consent was obtained under the ethics approval from the SingHealth CRIB Committee.

### Clinical samples

Women with euploidy and Down syndrome (DS) pregnancies who attended KK Women's and Children's Hospital, Singapore, were recruited.

Chorionic villus samples from subjects carrying a normal or DS fetus at the first or second trimesters of pregnancy were collected by chorinic villus sampling (CVS). Placenta villi samples (fetal side) from DS fetuses were collected from termination of pregnancy (TOP). All tissue samples were washed with diethylpyrocarbonate (Sigma-Aldrich, USA) treated water. For DNA analysis, tissues were stored at −80°C. For RNA analysis, tissues were incubated with RNA*later* (Life Technologies, USA) at 4°C overnight, and then stored at −80°C. Genomic DNA extraction from tissues was performed with QIAamp DNA Mini Kit (QIAGEN GmbH, Germany), according to manufacturer's instructions. Total RNA was extracted from frozen tissues using TRIZOL protocol (Life Technologies).

### Reduced representation bisulfite sequencing (RRBS)

Six DNA samples from normal pregnancies and 11 samples from pregnancies carrying DS fetuses were chosen for DNA methylation analysis by RRBS (Table S1). Briefly, 1–5 µg of high molecular weight (>10 kb) genomic DNA was used for each library preparation. Each DNA sample was sequentially digested by MspI (New England Biolabs, USA) (150 Units, two hours, 37°C) and Taq^α^I (New England Biolabs) (150 Units, two hours, 65°C). The digested product was purified with the QIAquick PCR Purification Kit (QIAGEN GmbH), and was end-repaired, 3′-end-adenylated, and adapter-ligated using ChIP-Seq Sample Preparation Kit (Illumina, USA). Illumina's RRBS for Methylation Analysis protocol was followed, except that 10 µL of the methylation adapter oligonucleotides were used and the ligation was performed for 15 min at 20°C in the adapter-ligation step. Two different sizes of fragments (150–197 bp and 207–230 bp) were selected by gel electrophoresis with a 3% agarose gel. The purified fragments were then bisulfite treated using the EZ DNA Methylation-Gold Kit (Zymo Research, USA). The converted DNA was amplified using HotStarTaq DNA Polymerase Kit (QIAGEN GmbH), with 1× reaction buffer, 1.5 mM of additional MgCl_2_, 300 µM of dNTP mix, 500 nM each of PCR primer PE 1.0 and 2.0, and 2.5 U of HotStarTaq DNA polymerase. The thermocycling condition was 15 min at 94°C for heat activation, and 8–12 cycles of 20 sec at 94°C, 30 sec at 65°C and 30 sec at 72°C, followed by a 5 min final extension at 72°C. The amplified fragments were purified by gel electrophoresis and further quantified by the Agilent 2100 Bioanalyzer (Agilent Technologies, USA). Each DNA library was analyzed by two lanes of paired-end sequencing (2×36 bp) read on an Illumina Genome Analyzer II_x_. Sequencing data were deposited into the GEO database with accession numbers GSE42144.

The paired-end 36 bp reads were filtered based on their Phred scores, using a cutoff of 30 which indicates a base calling error probability of 0.001. All reads were then converted in silico based on the C/G base count ratios. Two reference genomes were created, obtained by either converting all cytosine to thymines (C2T converted genome), or all guanines to adenosines (G2A converted genome). The converted reads were aligned to both genomes using the Bowtie program [48]. Bisulfite conversion rate was calculated by:
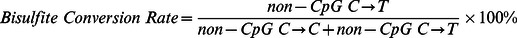
Where non-CpG C→T indicates successful conversion of C to T in non-CpG sites, and non-CpG C→C indicates failed conversion of C to T in non-CpG sites.

Polymorphisms overlapping with CpGs may introduce abnormalities. In this regard, CpG sites with percentage of dinucleotide ‘XY’ other than ‘CG’ or ‘TG’ greater than 20% of all reads were deemed to be polymorphic for the sample and were excluded for further analysis.

### Differential DNA methylation analysis

Differential DNA methylation between normal and DS samples were analyzed at single CpG level and at genomic region (CGI and promoters) levels. A total of 1,562,872 CpGs covered in at least 3 normal samples and at least 6 DS samples were used for further analysis. CpGs on the chromosomes X and Y were excluded. A CpG was considered as differentially methylated when 1) methylation difference between average DS and average normal samples was at least 10%; and 2) *p*<0.05, Wilcoxon rank-sum test, two-sided. For genomic regions, at least 6 CpGs in each genomic region were required. A genomic region was considered as differentially methylated when 1) methylation difference between average DS and average normal samples was at least 10%; and 2) *p*<0.05, Wilcoxon rank-sum test, two-sided.

Probability density function (PDF) for methylation differences between DS and normal samples were calculated and plotted with the R package.

### mRNA Sequencing (mRNA-SEQ)

Five RNA samples from normal pregnancies and 4 samples from pregnancies carrying DS fetuses were chosen for mRNA-seq analysis (Table S1). Briefly, 2–5 µg of total RNA was used for each library preparation. Each RNA sample was treated with DNase I (Life Technologies). Messenger RNA purification and fragmentation, complementary DNA synthesis, end-repair, 3′-end-adenylation, and adapter-ligation were performed using Illumina's mRNA-Seq Sample Preparation Kit. Manufacturer's instructions were followed, except that the SuperScript III First-Strand Synthesis SuperMix (Life Technologies) was used for first strand cDNA synthesis. Adapter-ligated cDNA fragments were size-selected using a 3% agarose gel (200±25 bp). The DNA samples were then amplified by PCR for 15–16 cycles. The PCR products were purified using 3% agarose gels and further quantified by the Agilent 2100 Bioanalyzer (Agilent Technologies). Each library was analyzed by one lane of either 36 bp single read or 2×36 bp paired-end sequencing on an Illumina Genome Analyzer II_x_.

### Differential gene expression analysis

RNA-Seq data were analyzed using Illumina RNA-Seq pipeline, CASAVA software version 1.7. The high quality reads were aligned step-wise to three reference files, mitochondrial DNA (chrM) that makes up the contaminant reference, hg19 genome assembly, and splice junction set created using the refFlat file, using default parameters. All the reference sequences were downloaded from UCSC website (http://hgdownload.cse.ucsc.edu/goldenPath/hg19/chromosomes/).

The expression level for each gene was represented by the reads per kilobase per million mapped reads (RPKM) value, using the formula below:
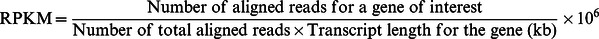
Average RPKM values for each gene in each sample group (normal and DS) were calculated. When the average RPKM for a gene is less than 0.5, the value was set as 0.5. A gene was considered to be differentially expressed between normal and DS samples when: 1) Binomial test with a Benjamini-Hochberg corrected *p* value of less than 0.01; and 2) the ratio of (Average DS/Average normal) ≥1.25 or ≤0.8. We used the R package to calculate the PDF distributions for various gene groups with regard to the expression changes represented by log_2_(Average DS/Average normal).

### Statistical analysis for genes overlapping between this study and the Kerkel study

Given that only 108 of the 14,000 (0.77%) genes and 598 out of 16,821 (3.6%) genes were significantly differentially methylated in the Kerkel study and this study respectively, three out of the nine genes sharing similar differential methylation are statistically significant (*p*<1.8×10^−9^) for three or more genes shared between two datasets, based on a combined probability of 0.77%×3.6% under the null hypothesis that the occurrence of differentially methylated genes were independent in the two tissues.

### DNA methylation validation by EpiTYPER assays

Gestational age matched normal (n = 14, gestational age: 17.41±3.77 weeks) and DS (n = 17, gestational age: 17.70±3.77 weeks) placenta villi samples were used for differential

DNA methylation validation using the EpiTYPER assays. Unless specified, all reagents and equipment were from Sequenom (San Diego, California, USA). Briefly, bisulfite conversion was performed on 1 µg genomic DNA with the EZ DNA Methylation-Gold Kit (Zymo Research, USA). The converted DNA was amplified using HotStarTaq DNA Polymerase Kit (QIAGEN GmbH), with 1× reaction buffer, 1.5 or 2.5 mM of additional MgCl_2_, 200 µM of dNTP mix, 200 nM each of forward and reverse primers (Table S7), and 1 unit of HotStarTaq DNA polymerase. The thermocycling condition was 15 min at 94°C for heat activation, and 50 cycles of 20 sec at 94°C, 30 sec at 50 or 55°C and 1 min at 72°C, followed by a 3 min final extension at 72°C. The PCR products were then treated with shrimp alkaline phosphatase, and subsequently with the T-cleavage transcription/RNase A cocktail from EpiTYPER Reagent Kit (Sequenom). The reaction products were subjected to conditioning with Clean Resin, and the fragments were analyzed by the MassARRAY system. Data were analyzed using EpiTYPER 1.2 software (Sequenom). DNA methylation level for each sample was determined by averaging all analyzed CpGs within the target amplicon.

### Quantitative real-time PCR validation

Gestational age matched normal (n = 8, gestational age: 19.18±3.56 weeks) and DS (n = 10, gestational age: 18.37±2.70 weeks) placenta villi samples were used for differential gene expression validation. All reagents and equipment involved were from Life Technologies. DNase I treated total RNA samples (0.5 to 1 µg total RNA) were subject to first strand DNA synthesis by SuperScript III First-Strand Synthesis SuperMix Kit. Quantitative real-time PCR was performed with Applied Biosystems 7900HT Fast Real-time PCR system with 384-well block module. Each reaction contained 1× Power SYBR Green Master Mix, 100 nM each of forward and reverse primers (Table S8) and cDNA template equivalent to 18.2 ng of total RNA in a 10 µL reaction. The thermocycling condition was 10 min at 95°C, and 40 cycles of 15 sec at 95°C and 1 min at 60°C, followed by melting curve analysis. Duplicate reactions were performed for each assay, and the average Ct value was obtained using SDS version 2.3 software. GAPDH was used for normalization, with the following formula:
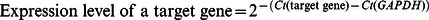



## Supporting Information

Figure S1 RRBS coverage, fragment size and principal component analysis (PCA). (**A**) An example for number of CpG sites with different minimum sequencing depths. Numbers of CpGs sites covered at ≥5 and ≥10 are provided for each sample in Table S1. (**B**) Fragment size distribution for a representative library. (C) PCA results. N.F: normal female, N.M: normal male, T.F: DS female, T.M: DS male.
(DOCX)

Figure S2 RRBS technical replicates and comparison with published results. (**A**) Technical replicates for one sample. (**B–C**) Comparison between published results (Eckman-Scholz *et al.*) and this study in normal (**B**) and DS (**C**) samples for 2,894 CpGs analyzed by both methods.
(DOCX)

Figure S3 Distributions for individual CpG methylation. (**A**) all CpGs; (**B**) CpGs in promoter regions; (**C**) CpGs in TTRs; (**D**) CpGs in intragenic regions; (**E**) CpGs in intergenic regions. The methylation level for each CpG was calculated based on the average values for normal and DS samples, respectively.
(DOCX)

Figure S4 Inter-individual variability for CpGs. Only CpGs with average methylation between 30–70% for the five normal samples with male fetuses were used since these CpGs were most variable. Such CpGs were further selected based on minimum sequencing depths of 10, 20 or 50. Most CpGs had standard deviations among the five normal samples at lower than 10%. As expected, with increasing cut-off of depth the variability decreased, suggesting at least some variability was derived from sequencing depth.
(DOCX)

Figure S5 Differentially methylated genes were shared between DS peripheral blood leukocytes and placenta villi samples. (**A**) Comparison of DNA methylation for seven genes determined by RRBS and EpiTYPER. Different sample sets were used for the two methods. (**B**) Three genes (*TCF7*, *FAM62C* and *CPT1B*) were similarly differentially methylated in leukocytes (Kerkel *et al.*) and placenta villi samples (this study) (***: *p*<0.001, t-test). Error bars represent standard deviations. RRBS: normal n = 6, DS n = 11. EpiTYPER: normal n = 14, DS n = 17.
(DOCX)

Figure S6 Genes with hypermethylated promoters are associated with expression down-regulation in DS. (**A**) Promoter hypermethylation in DS samples. (**B**) Down-regulation of gene expression in DS samples. *: *p*<0.05, **: *p*<0.01, ***: *p*<0.001, t-test, two-sided. Error bars represent standard deviations. EpiTYPER: normal n = 14, DS n = 17. Gene expression: normal n = 8, DS n = 10.
(DOCX)

Figure S7 Quantitative real-time PCR validation of expression changes for (**A**) *TET1*, (**B**) *TET2*, (**C**) *TET3* and (**D**) *REST*. *: *p*<0.05, **: *p*<0.01. t-test, two-sided. Error bars represent standard deviations. Sample size: normal n = 8, DS n = 10.
(DOCX)

Figure S8 Pathway and network analyses for genes with differentially methylated promoters in DS. Data were analyzed by MetaCore (http://www.genego.com, GeneGo Inc.). (**A**) Top 10 GeneGo pathway maps; (**B**) The network for “Immune response_Lectin induced complement pathway”; (**C**) The network for “Neurophysiological process_Dopamine D2 receptor signaling in CNS”; (**D**) The network for “Cytoskeleton remodeling_Neurofilaments; (**E**) Top 10 GeneGo process networks. In (**B**), (**C**) and (**D**), the genes with differentially methylated promoter in DS were marked with red thermometer shape.
(DOCX)

Figure S9 Cellular localizations of REST/NRSF target genes.
(DOCX)

Table S1 Sample and sequencing information.
(DOCX)

Table S2 Frequencies of differentially methylated CpGs in different genomic regions.
(DOCX)

Table S3 Sample information for EpiTYPER and quantitative real-time PCR validations.
(DOCX)

Table S4 Expression levels of chr21 and other selected genes.
(PDF)

Table S5 Differentiated methylated genes with causal association to DS.
(DOCX)

Table S6 Top 10 transcription factors whose target genes were either enriched or depleted for differentially methylated promoters in DS.
(DOCX)

Table S7 EpiTYPER assays for DNA methylation validation.
(DOCX)

Table S8 Quantitative real-time PCR assays for gene expression validation.
(DOCX)
