# Supplementary figures and images for: Global DNA Hypermethylation in Down Syndrome Placenta
Source: PLoS Genet. 2013 Jun 6;9(6):e1003515. doi: 10.1371/journal.pgen.1003515 (PMC3675012; doi:10.1371/journal.pgen.1003515)

**A**

**
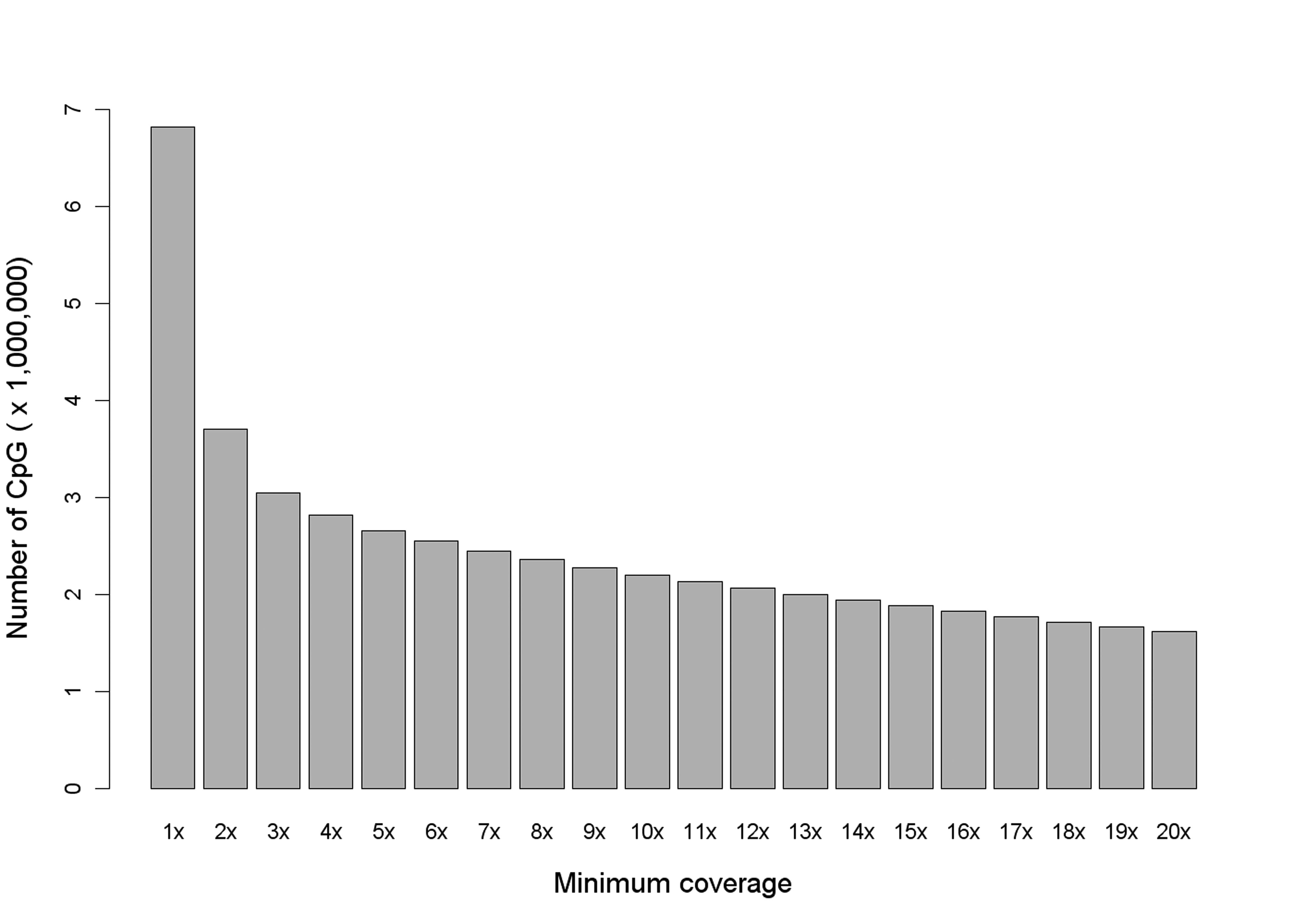
**

**B**


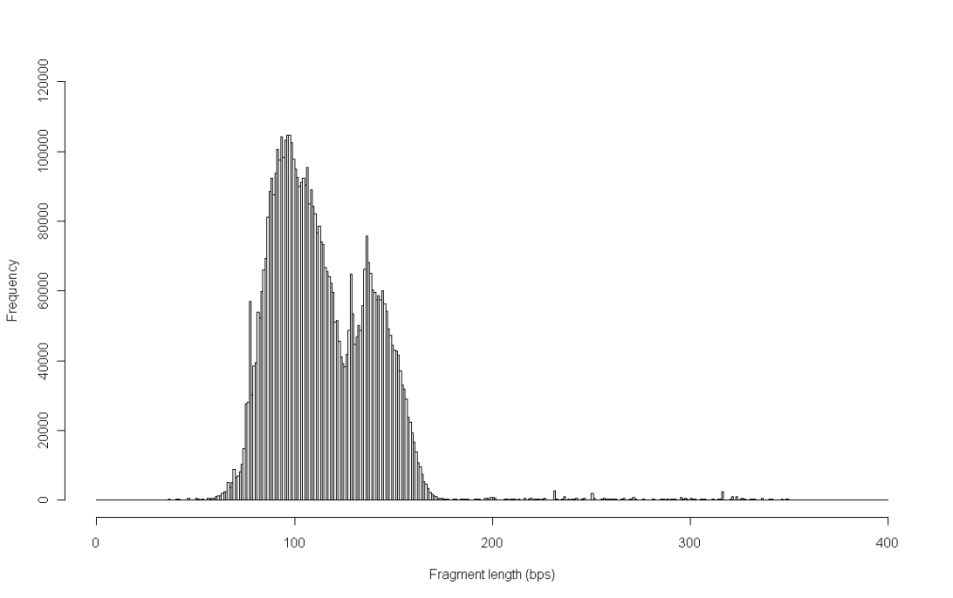


**C**

**
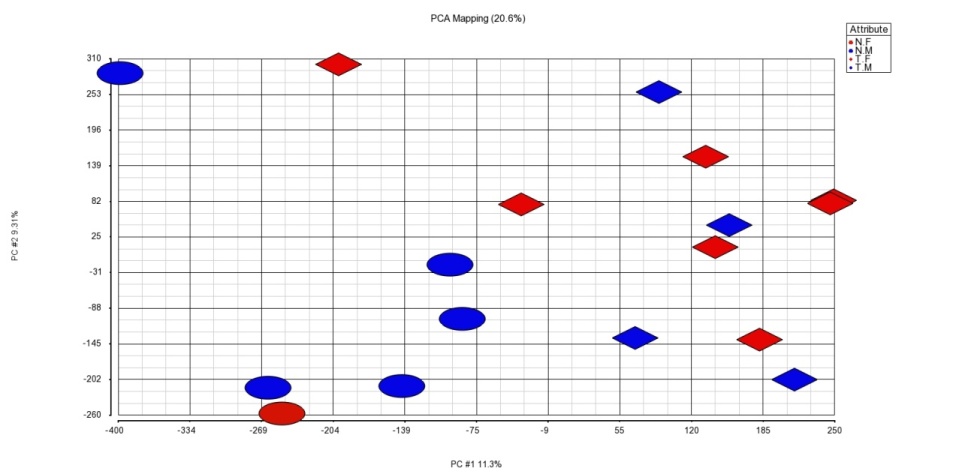
**

Supplement: Figure S1 — RRBS coverage, fragment size and principal component analysis (PCA). (A) An example for number of CpG sites with different minimum sequencing depths. Numbers of CpGs sites covered at ≥5 and ≥10 are provided for each sample in Table S1. (B) Fragment size distribution for a representative library. (C) PCA results. N.F: normal female, N.M: normal male, T.F: DS female, T.M: DS male. (DOCX) [file pgen.1003515.s001.docx]

**A**

**
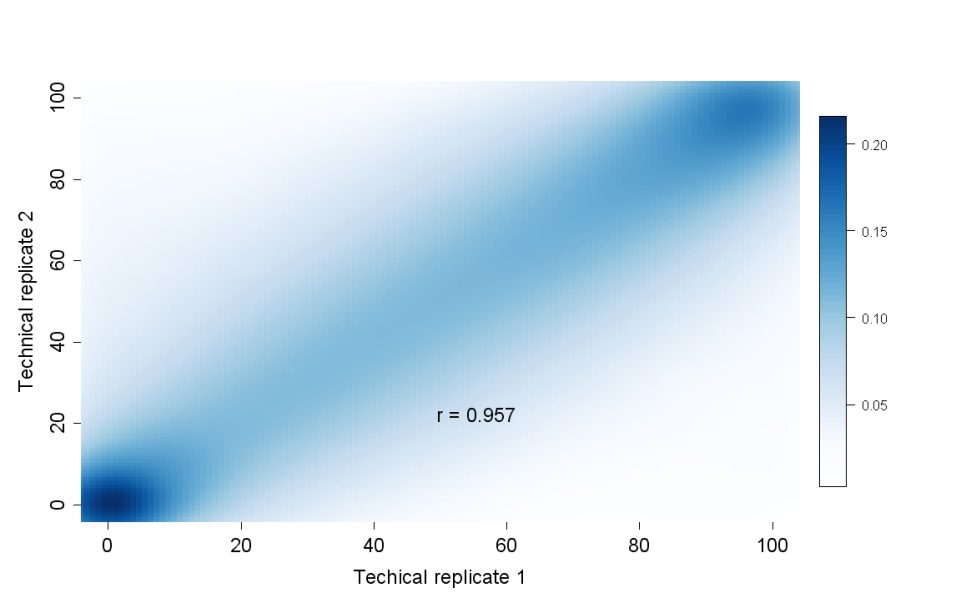
**

**B**

**
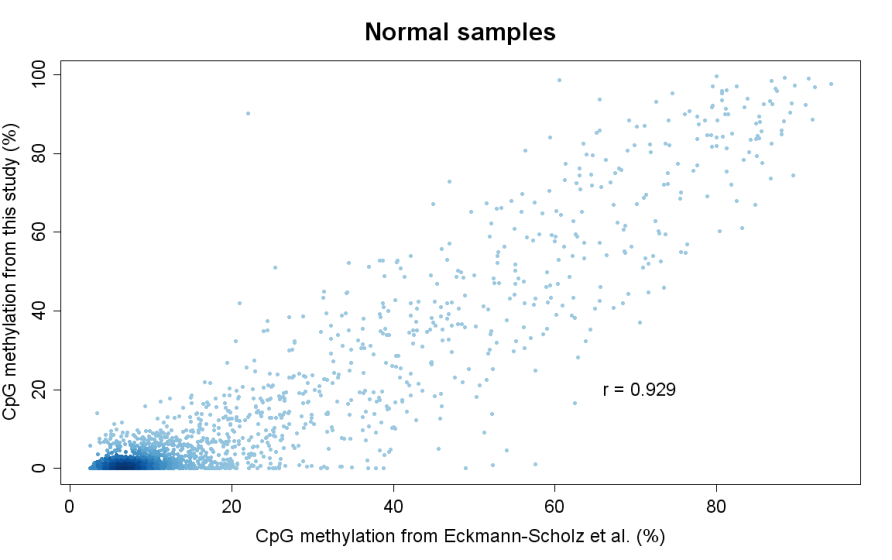
**

**C**

**
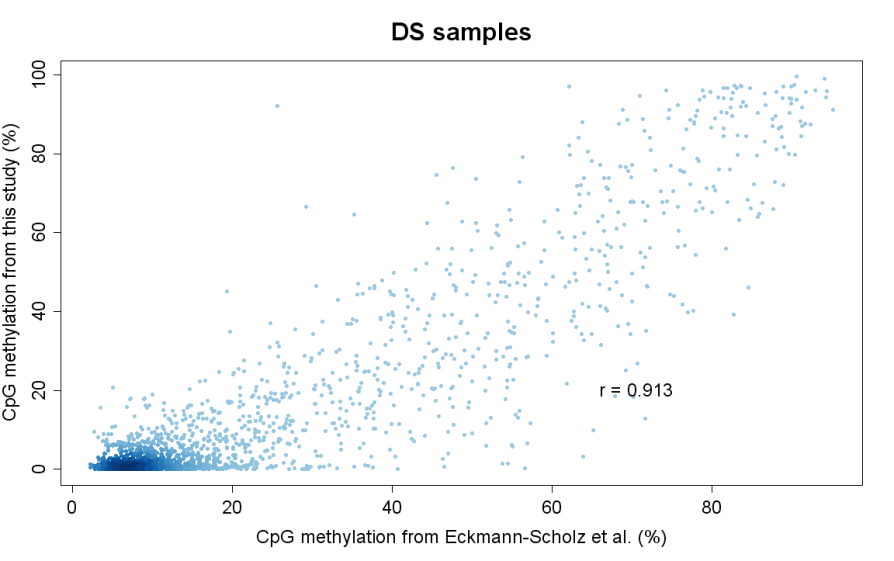
**

Supplement: Figure S2 — RRBS technical replicates and comparison with published results. (A) Technical replicates for one sample. (B–C) Comparison between published results (Eckman-Scholz et al.) and this study in normal (B) and DS (C) samples for 2,894 CpGs analyzed by both methods. (DOCX) [file pgen.1003515.s002.docx]

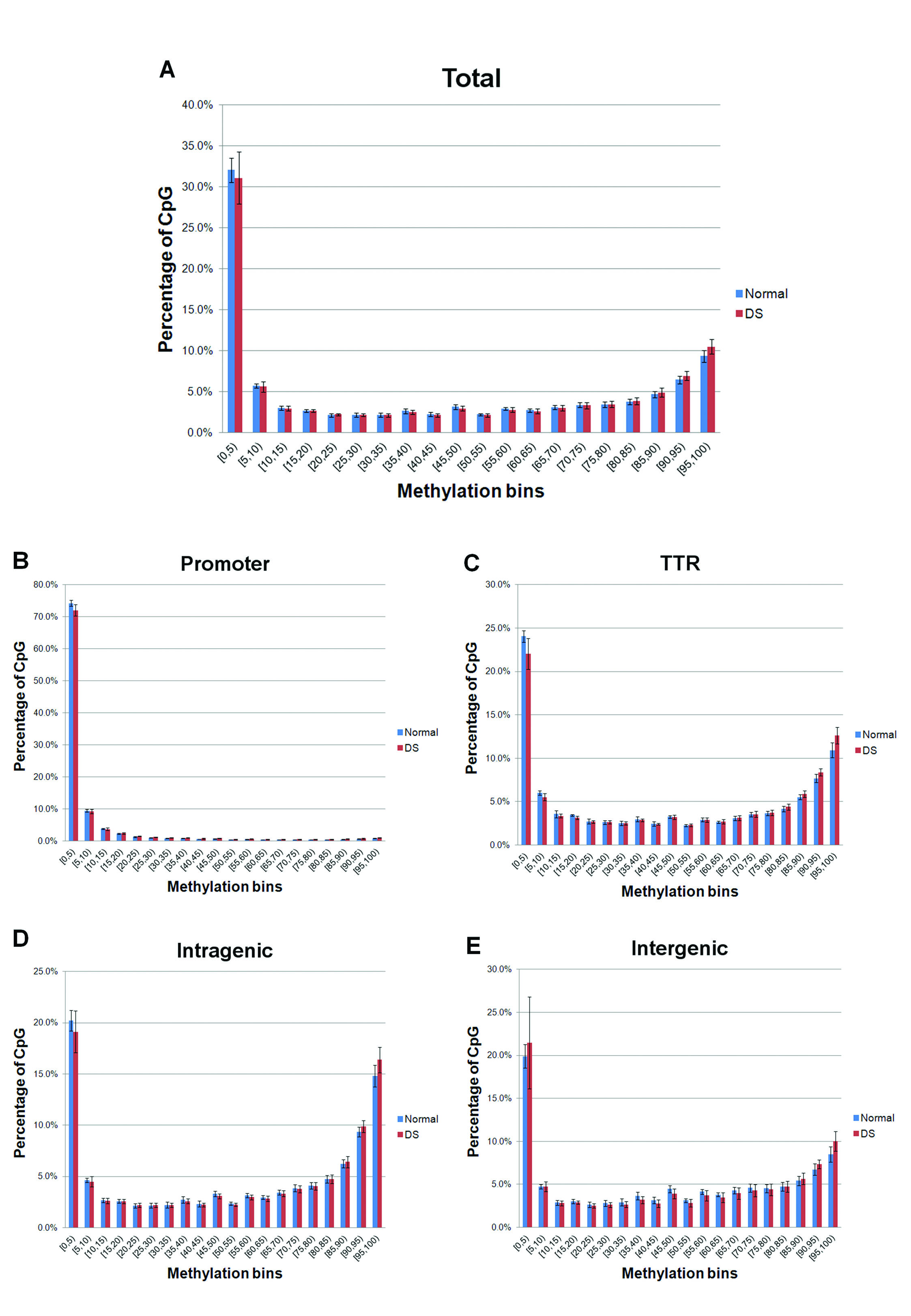

Supplement: Figure S3 — Distributions for individual CpG methylation. (A) all CpGs; (B) CpGs in promoter regions; (C) CpGs in TTRs; (D) CpGs in intragenic regions; (E) CpGs in intergenic regions. The methylation level for each CpG was calculated based on the average values for normal and DS samples, respectively. (DOCX) [file pgen.1003515.s003.docx]

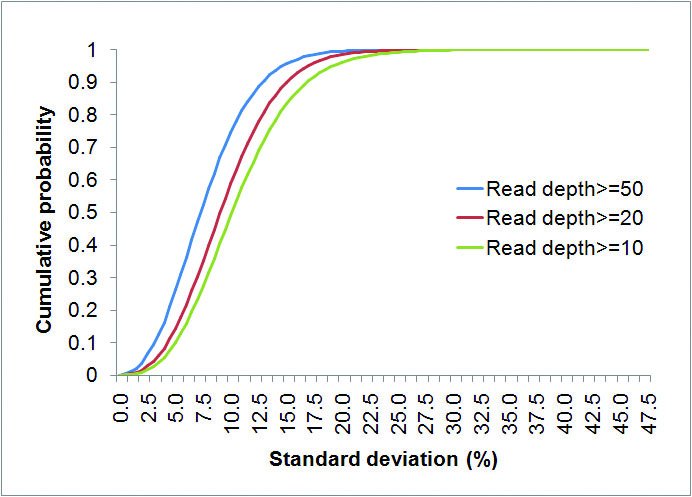

Supplement: Figure S4 — Inter-individual variability for CpGs. Only CpGs with average methylation between 30–70% for the five normal samples with male fetuses were used since these CpGs were most variable. Such CpGs were further selected based on minimum sequencing depths of 10, 20 or 50. Most CpGs had standard deviations among the five normal samples at lower than 10%. As expected, with increasing cut-off of depth the variability decreased, suggesting at least some variability was derived from sequencing depth. (DOCX) [file pgen.1003515.s004.docx]

**A**


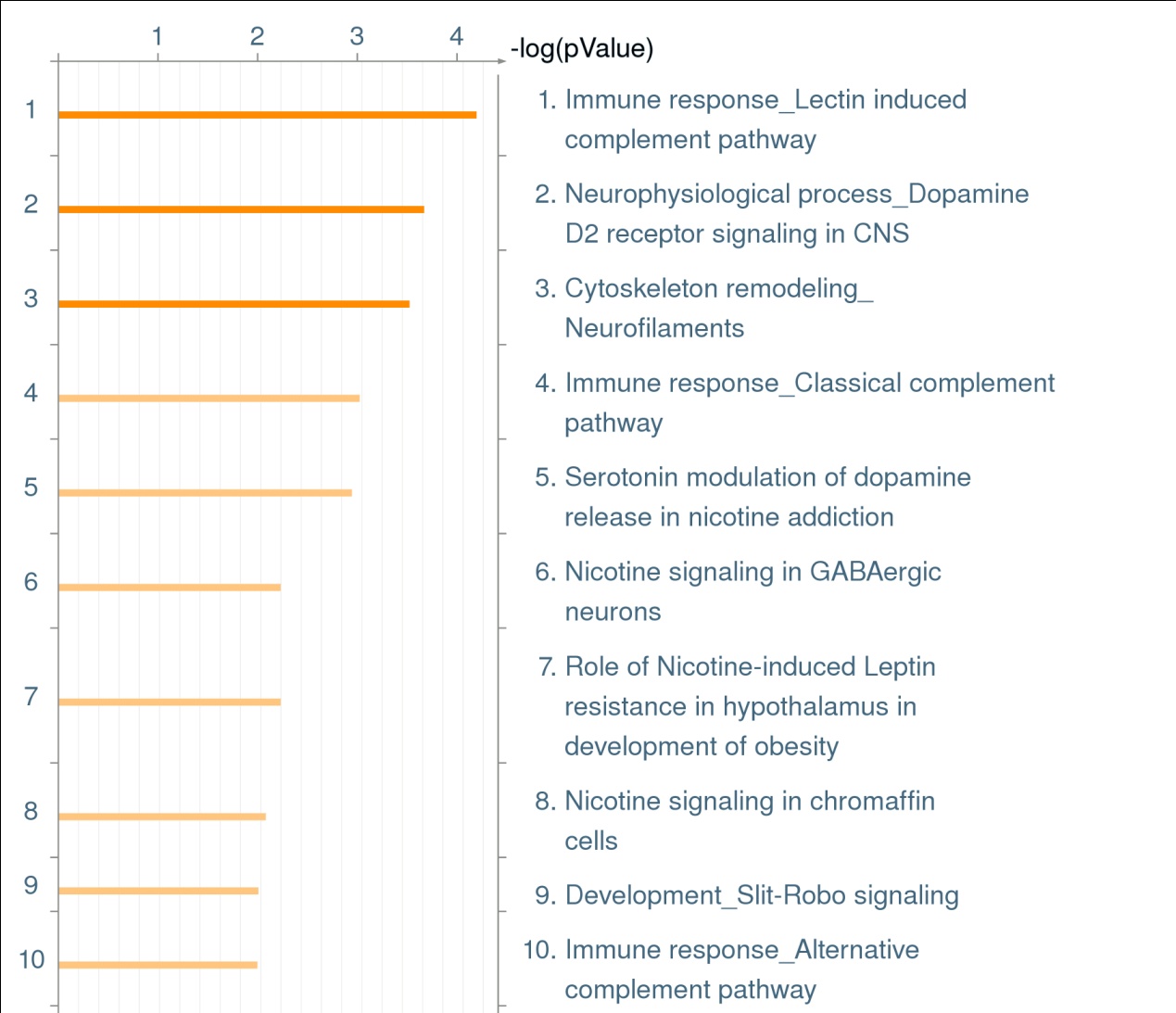


**B**

**
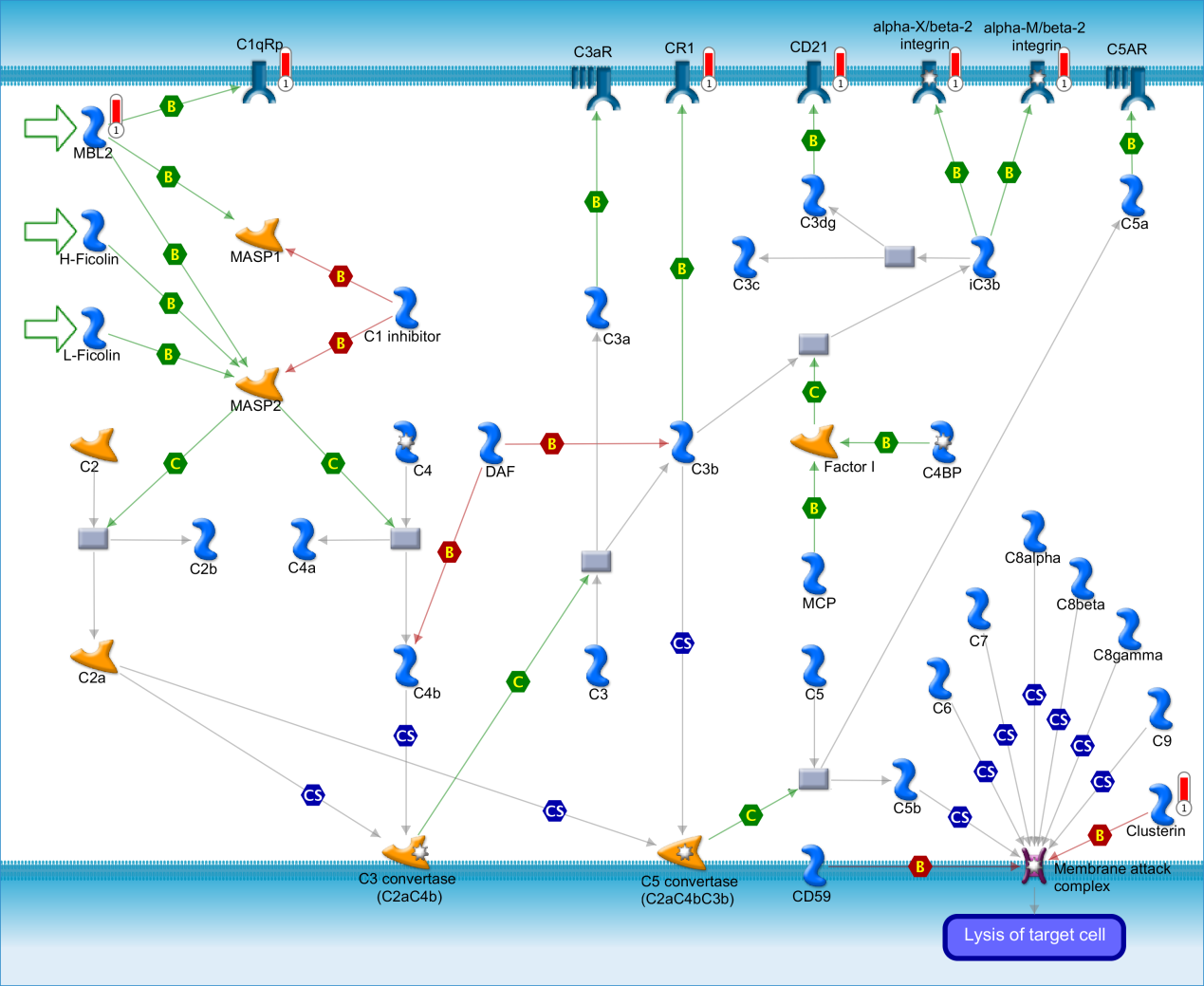
**

**C**

**
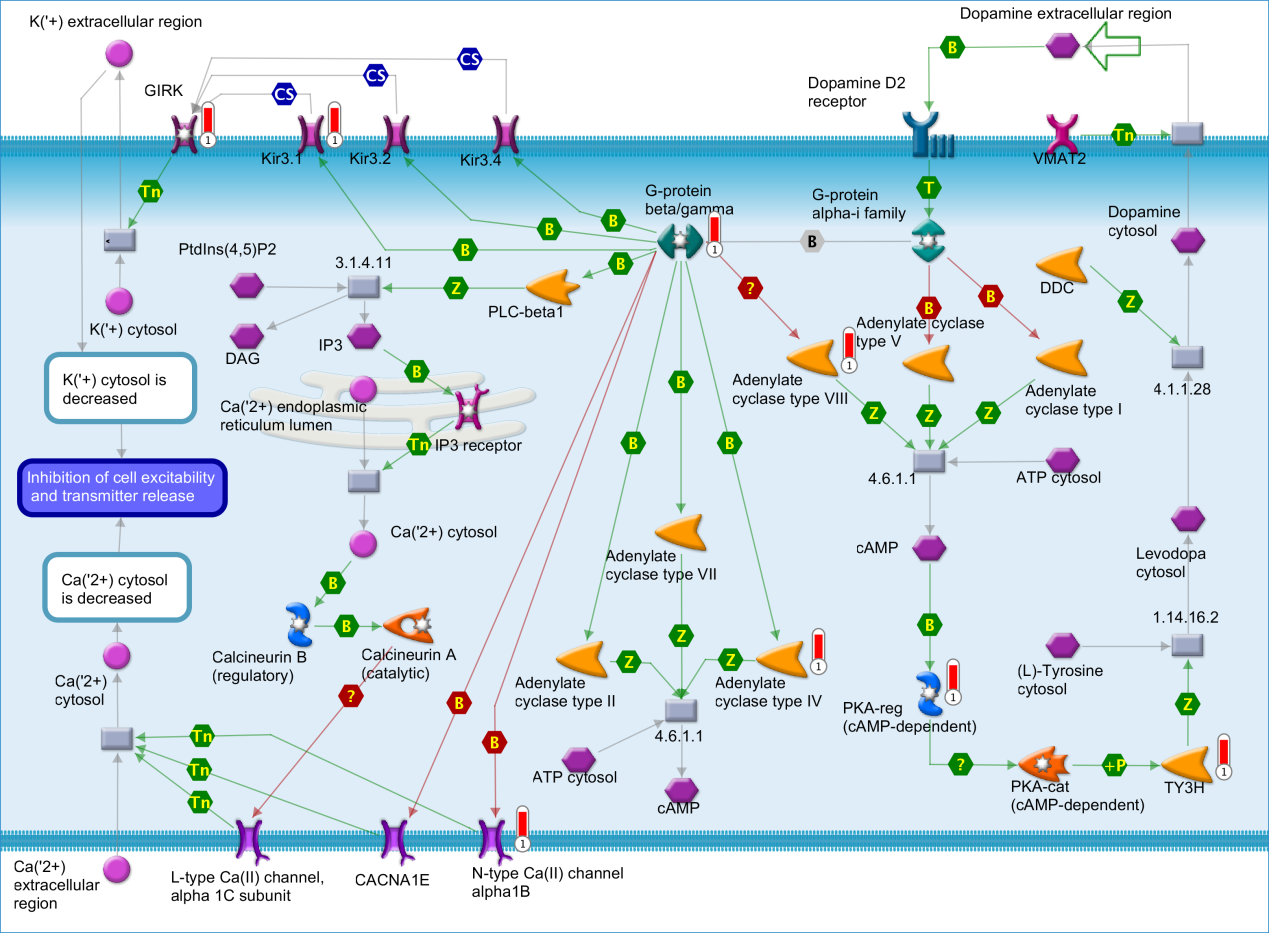
**

**D**


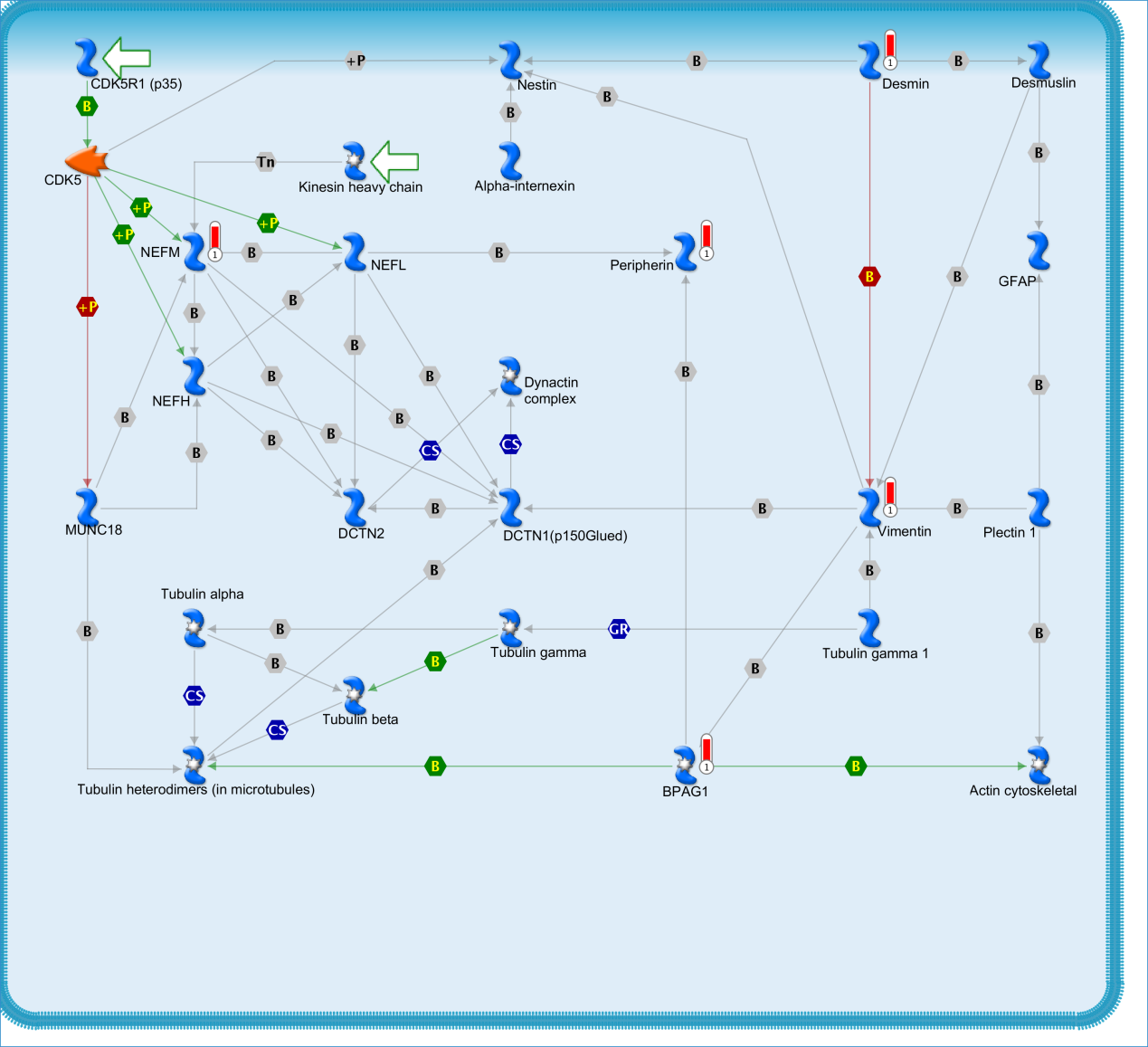


**E**


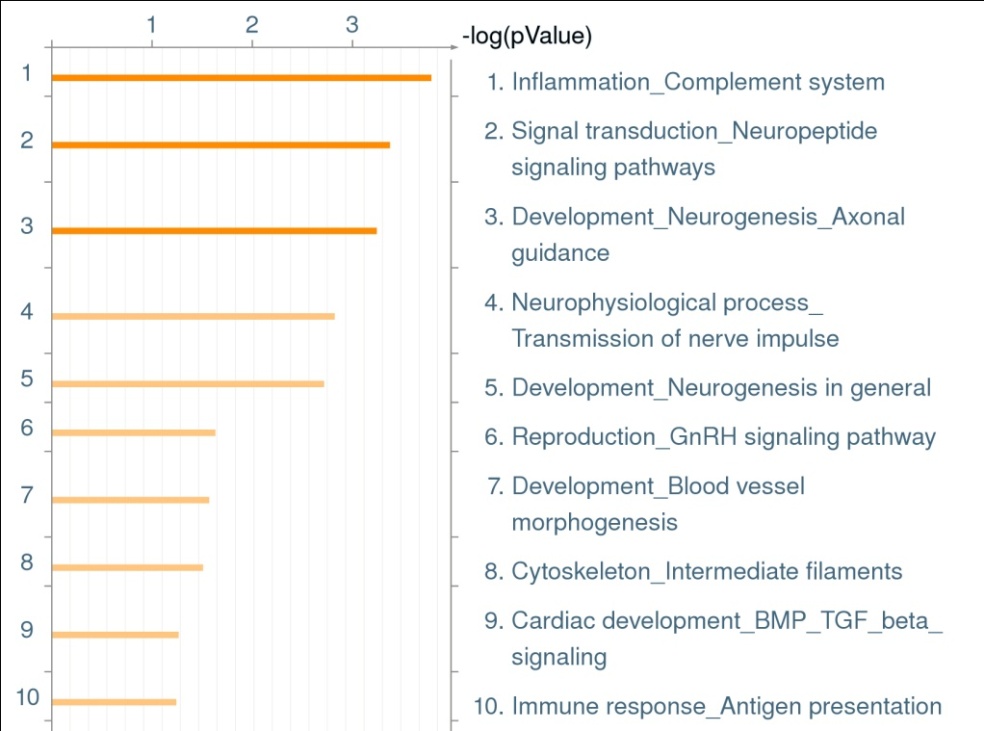

Supplement: Figure S8 — Pathway and network analyses for genes with differentially methylated promoters in DS. Data were analyzed by MetaCore (http://www.genego.com, GeneGo Inc.). (A) Top 10 GeneGo pathway maps; (B) The network for “Immune response_Lectin induced complement pathway”; (C) The network for “Neurophysiological process_Dopamine D2 receptor signaling in CNS”; (D) The network for “Cytoskeleton remodeling_Neurofilaments; (E) Top 10 GeneGo process networks. In (B), (C) and (D), the genes with differentially methylated promoter in DS were marked with red thermometer shape. (DOCX) [file pgen.1003515.s008.docx]

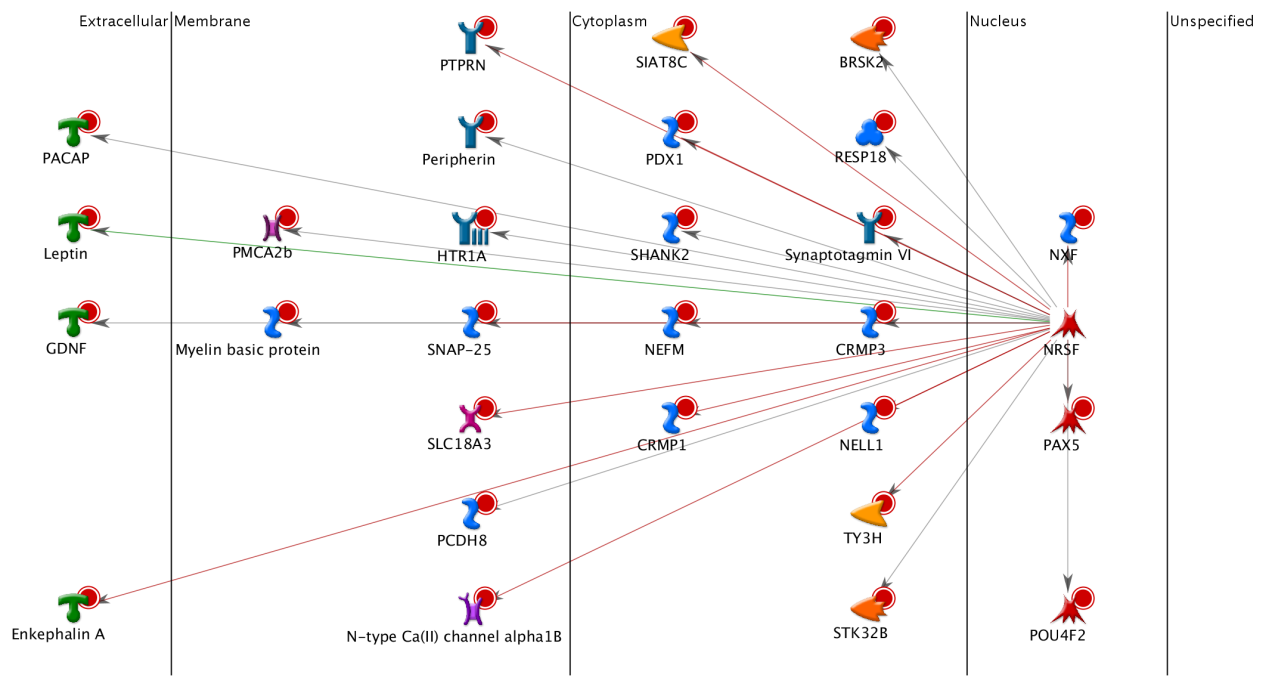

Supplement: Figure S9 — Cellular localizations of REST/NRSF target genes. (DOCX) [file pgen.1003515.s009.docx]
