# Supplementary material for: Global DNA Hypermethylation in Down Syndrome Placenta
Source: PLoS Genet. 2013 Jun 6;9(6):e1003515. doi: 10.1371/journal.pgen.1003515 (PMC3675012; doi:10.1371/journal.pgen.1003515)
Supplement: Table S1 — Sample and sequencing information. (DOCX) [file pgen.1003515.s010.docx]

**Supplemental Table 1** Sample and sequencing information.

| Patient No | Description | Analysis | Mother Ethnicity | Fetal Gender | Gestation Week | RIN (RNA only) | Pass Filtered Read # | Aligned Read # | # of CpG covered ≥ 5 | # of CpG covered ≥ 10 | Average Conversion Rate |
| --- | --- | --- | --- | --- | --- | --- | --- | --- | --- | --- | --- |
| N1 | Normal | RRBS | Asian | M | 13 5/7 | NA | 27,165,626 | 16,753,120 | 1,590,925 | 1,334,390 | 99.32% |
| N2 | Normal | RRBS | Chinese | M | 12 3/7 | NA | 32,423,718 | 17,888,562 | 2,119,114 | 1,668,848 | 99.32% |
| N3 | Normal | RRBS | Chinese | M | 13 | NA | 36,218,096 | 20,351,034 | 2,357,402 | 1,906,340 | 99.33% |
| N4 | Normal | RRBS | Chinese | M | 11 6/7 | NA | 49,466,043 | 28,357,412 | 2,467,049 | 2,062,492 | 99.10% |
| N5 | Normal | RRBS | Chinese | M | 12 5/7 | NA | 48,223,157 | 28,784,365 | 2,657,349 | 2,200,928 | 99.16% |
| N6 | Normal | RRBS | Asian | F | 14 | NA | 32,456,830 | 18,687,265 | 2,412,422 | 2,010,234 | 99.29% |
| T1 | DS | RRBS | Chinese | F | 14 1/7 | NA | 20,880,016 | 13,102,467 | 1,429,539 | 1,137,494 | 99.14% |
| T2 | DS | RRBS | NA | M | 13 1/7 | NA | 28,457,877 | 17,904,741 | 1,700,132 | 1,389,531 | 99.35% |
| T3 | DS | RRBS | Chinese | F | 14 6/7 | NA | 35,013,862 | 20,559,170 | 1,917,248 | 1,602,934 | 99.32% |
| T4 | DS | RRBS | Chinese | F | 13 6/7 | NA | 37,912,282 | 20,752,720 | 1,970,150 | 1,618,385 | 99.33% |
| T5 | DS | RRBS | Chinese | F | 14 3/7 | NA | 40,340,161 | 23,530,943 | 1,706,492 | 1,438,991 | 99.30% |
| T6 | DS | RRBS | Chinese | F | 15 5/7 | NA | 42,674,695 | 19,755,607 | 2,248,703 | 1,849,714 | 99.05% |
| T7 | DS | RRBS | Chinese | F | 18 3/7 | NA | 39,219,943 | 20,975,033 | 1,971,596 | 1,647,728 | 99.15% |
| T8 | DS | RRBS | Chinese | F | 15 6/7 | NA | 50,148,095 | 29,351,241 | 2,077,168 | 1,701,734 | 99.01% |
| T9 | DS | RRBS | Chinese | M | 16 4/7 | NA | 49,548,992 | 25,642,700 | 1,621,855 | 1,299,896 | 99.02% |
| T10 | DS | RRBS | Chinese | M | 21 4/7 | NA | 32,845,049 | 19,285,051 | 2,536,447 | 2,009,872 | 99.30% |
| T11 | DS | RRBS | Chinese | M | 19 | NA | 34,699,496 | 21,345,214 | 2,276,068 | 1,836,570 | 99.37% |
| N5 | Normal | mRNA-seq | Chinese | M | 12 5/7 | 8.2 | 41,872,173 | 29,434,733 | NA | NA | NA |
| N7 | Normal | mRNA-seq | Chinese | M | 13 | 9 | 19,968,401 | 13,221,738 | NA | NA | NA |
| N8 | Normal | mRNA-seq | Chinese | F | 13 6/7 | 8.2 | 42,021,819 | 28,886,664 | NA | NA | NA |
| N9 | Normal | mRNA-seq | Chinese | F | 13 3/7 | 8.2 | 39,535,789 | 27,021,552 | NA | NA | NA |
| N10 | Normal | mRNA-seq | Chinese | M | 14 4/7 | 8.5 | 40,264,585 | 27,574,102 | NA | NA | NA |
| T3 | DS | mRNA-seq | Chinese | F | 14 6/7 | 8.2 | 17,785,316 | 11,971,826 | NA | NA | NA |
| T6 | DS | mRNA-seq | Chinese | F | 15 5/7 | 7.4 | 37,381,545 | 25,767,702 | NA | NA | NA |
| T12 | DS | mRNA-seq | Chinese | F | 13 6/7 | 9.5 | 17,833,455 | 12,103,982 | NA | NA | NA |
| T13 | DS | mRNA-seq | Chinese | M | 15 2/7 | 7.5 | 31,565,862 | 23,834,613 | NA | NA | NA |
