# Supplementary material for: Global DNA Hypermethylation in Down Syndrome Placenta
Source: PLoS Genet. 2013 Jun 6;9(6):e1003515. doi: 10.1371/journal.pgen.1003515 (PMC3675012; doi:10.1371/journal.pgen.1003515)
Supplement: Table S2 — Frequencies of differentially methylated CpGs in different genomic regions. (DOCX) [file pgen.1003515.s011.docx]

**Supplemental Table 2** Frequencies of differentially methylated CpGs in different genomic regions

| CpG | Total number of CpGs^a^ | DS > Normal^b^ | Normal > DS | Ratio^c^ |
| --- | --- | --- | --- | --- |
| Promoter (All) | 503,974 | 18,937 (3.8%) | 721 (0.14%) | 26.3 |
| Promoter (CGI) | 414,826 | 14,500 (3.5%) | 258 (0.06%) | 56.2 |
| Promoter (Non-CGI) | 89,148 | 4,437 (5.0%) | 463 (0.52%) | 9.58 |
| TTR | 38,550 | 3,415 (8.9%) | 260 (0.67%) | 13.1 |
| Intragenic | 610,084 | 38,436 (6.3%) | 5,298 (0.87%) | 7.25 |
| Intergenic | 410,264 | 33,944 (8.3%) | 3,862 (0.94%) | 8.79 |
| Total | 1,562,872 | 94,732 (5.5%) | 10,141 (0.53%) | 9.34 |
| ^a^CpGs with sequencing depth ≥10 in at least 3 normal and 6 DS samples. ^b^CpGs with (average DS samples methylation – average normal samples methylation ≥10%) and  *P* < 0.05 (Wilcoxon rank-sum test, two-sided). ^c^Ratio of CpGs more methylated in DS vs CpGs more methylated in normal samples. | | | | |
