# Supplementary material for: Global DNA Hypermethylation in Down Syndrome Placenta
Source: PLoS Genet. 2013 Jun 6;9(6):e1003515. doi: 10.1371/journal.pgen.1003515 (PMC3675012; doi:10.1371/journal.pgen.1003515)
Supplement: Table S3 — Sample information for EpiTYPER and quantitative real-time PCR validations. (DOCX) [file pgen.1003515.s012.docx]

**Supplemental Table 3** Sample information for EpiTYPER and qPCR validations.

| Patient No | Description | Sample Type | Analysis | Fetal Gender | Gestation Week |
| --- | --- | --- | --- | --- | --- |
| N11 | Normal | DNA | EpiTYPER | M | 12 5/7 |
| N12 | Normal | DNA | EpiTYPER | M | 13 5/7 |
| N13 | Normal | DNA | EpiTYPER | M | 13 6/7 |
| N14 | Normal | DNA | EpiTYPER | F | 13 6/7 |
| N15 | Normal | DNA | EpiTYPER | M | 14 |
| N16 | Normal | DNA | EpiTYPER | M | 14 4/7 |
| N17 | Normal | DNA | EpiTYPER | M | 16 3/7 |
| N18 | Normal | DNA | EpiTYPER | M | 17 6/7 |
| N19 | Normal | DNA | EpiTYPER | M | 18 3/7 |
| N20 | Normal | DNA | EpiTYPER | M | 20 |
| N21 | Normal | DNA | EpiTYPER | M | 20 4/7 |
| N22 | Normal | DNA | EpiTYPER | M | 20 5/7 |
| N23 | Normal | DNA | EpiTYPER | M | 23 |
| N24 | Normal | DNA | EpiTYPER | M | 24 |
| T14 | DS | DNA | EpiTYPER | M | 12 6/7 |
| T15 | DS | DNA | EpiTYPER | M | 13 |
| T16 | DS | DNA | EpiTYPER | M | 13 |
| T17 | DS | DNA | EpiTYPER | F | 13 6/7 |
| T18 | DS | DNA | EpiTYPER | M | 14 1/7 |
| T19 | DS | DNA | EpiTYPER | M | 14 3/7 |
| T20 | DS | DNA | EpiTYPER | M | 15 2/7 |
| T21 | DS | DNA | EpiTYPER | M | 16 6/7 |
| T22 | DS | DNA | EpiTYPER | M | 17 6/7 |
| T23 | DS | DNA | EpiTYPER | F | 18 3/7 |
| T24 | DS | DNA | EpiTYPER | M | 19 3/7 |
| T25 | DS | DNA | EpiTYPER | M | 19 5/7 |
| T26 | DS | DNA | EpiTYPER | M | 20 |
| T27 | DS | DNA | EpiTYPER | F | 20 5/7 |
| T28 | DS | DNA | EpiTYPER | M | 22 1/7 |
| T29 | DS | DNA | EpiTYPER | F | 22 6/7 |
| T30 | DS | DNA | EpiTYPER | M | 23 6/7 |
| N7 | Normal | RNA | qPCR | M | 13 |
| N17 | Normal | RNA | qPCR | M | 16 3/7 |
| N18 | Normal | RNA | qPCR | M | 17 6/7 |
| N19 | Normal | RNA | qPCR | M | 18 3/7 |
| N20 | Normal | RNA | qPCR | M | 20 |
| N22 | Normal | RNA | qPCR | M | 20 5/7 |
| N23 | Normal | RNA | qPCR | M | 23 |
| N24 | Normal | RNA | qPCR | M | 24 |
| T18 | DS | RNA | qPCR | M | 14 1/7 |
| T3 | DS | RNA | qPCR | F | 14 6/7 |
| T31 | DS | RNA | qPCR | M | 15 2/7 |
| T32 | DS | RNA | qPCR | F | 18 3/7 |
| T33 | DS | RNA | qPCR | M | 19 |
| T24 | DS | RNA | qPCR | M | 19 3/7 |
| T25 | DS | RNA | qPCR | M | 19 5/7 |
| T26 | DS | RNA | qPCR | M | 20 |
| T27 | DS | RNA | qPCR | F | 20 5/7 |
| T28 | DS | RNA | qPCR | M | 22 1/7 |
