# Supplementary material for: Global DNA Hypermethylation in Down Syndrome Placenta
Source: PLoS Genet. 2013 Jun 6;9(6):e1003515. doi: 10.1371/journal.pgen.1003515 (PMC3675012; doi:10.1371/journal.pgen.1003515)
Supplement: Table S4 — Expression levels of chr21 and other selected genes. (PDF) [file pgen.1003515.s013.pdf]

Category	Gene	Location	Average(Normal$_{RPKM}$)	Average(DS$_{RPKM}$)	(DS$_{RPKM}$/Normal$_{RPKM}$)	Corrected p-value
Chr21 genes	TPTE	Chr21:10906743-10990920	<0.5	<0.5	1.00	9.25E-01
Chr21 genes	BAGE	Chr21:11057796-11089753	<0.5	<0.5	1.00	1.00
Chr21 genes	BAGE4	Chr21:11097542-11097545	<0.5	<0.5	1.00	1.00
Chr21 genes	ANKRD30BP2	Chr21:14410487-14490569	<0.5	<0.5	1.00	1.00
Chr21 genes	MIR3156-3	Chr21:14778706-14778781	<0.5	<0.5	1.00	1.00
Chr21 genes	POTED	Chr21:14982498-15013906	<0.5	<0.5	1.00	1.00
Chr21 genes	C21orf15	Chr21:15215455-15220685	<0.5	<0.5	1.00	1.00
Chr21 genes	C21orf81	Chr21:15316096-15352765	<0.5	<0.5	1.00	1.00
Chr21 genes	LIPI	Chr21:15481137-15579254	<0.5	<0.5	1.00	1.00
Chr21 genes	RBM11	Chr21:15588466-15600691	<0.5	<0.5	1.00	4.58E-01
Chr21 genes	ABCC13	Chr21:15646120-15673690	<0.5	<0.5	1.00	1.00
Chr21 genes	HSPA13	Chr21:15743439-15755509	7.49	8.37	1.12	4.77E-01
Chr21 genes	SAMSN1	Chr21:15857549-15918664	1.26	1.68	1.34	4.08E-01
Chr21 genes	NRIP1	Chr21:16333556-16437126	6.40	6.06	0.95	1.12E-01
Chr21 genes	USP25	Chr21:17102496-17252377	14.65	16.61	1.13	5.80E-02
Chr21 genes	C21orf34	Chr21:17442842-17982094	<0.5	<0.5	1.00	1.00
Chr21 genes	MIR99A	Chr21:17911409-17911489	<0.5	<0.5	1.00	1.00
Chr21 genes	MIRLET7C	Chr21:17912148-17912231	<0.5	<0.5	1.00	1.00
Chr21 genes	MIR125B2	Chr21:17962557-17962644	<0.5	<0.5	1.00	1.00
Chr21 genes	CXADR	Chr21:18885330-18939264	<0.5	<0.5	1.00	1.00
Chr21 genes	BTG3	Chr21:18965970-18985268	30.25	40.40	1.34	8.90E-09
Chr21 genes	C21orf91	Chr21:19161285-19191703	4.98	6.61	1.33	1.06E-04
Chr21 genes	NCRNA00157	Chr21:19207989-19257925	<0.5	<0.5	1.00	8.23E-01
Chr21 genes	CHODL	Chr21:19617150-19639685	0.65	1.52	2.32	2.19E-04
Chr21 genes	TMPRSS15	Chr21:19641434-19775970	<0.5	<0.5	1.00	1.00
Chr21 genes	C21orf131	Chr21:22114914-22175426	<0.5	<0.5	1.00	1.00
Chr21 genes	NCAM2	Chr21:22370633-22911214	<0.5	<0.5	1.00	1.24E-01
Chr21 genes	NCRNA00158	Chr21:26758134-26804013	<0.5	<0.5	1.00	1.00
Chr21 genes	MIR155HG	Chr21:26934457-26947473	<0.5	<0.5	1.00	1.00
Chr21 genes	C21orf71	Chr21:26955088-26955536	<0.5	<0.5	1.00	1.00
Chr21 genes	MRPL39	Chr21:26957970-26979801	11.23	15.47	1.38	1.61E-03
Chr21 genes	JAM2	Chr21:27011589-27087235	13.28	12.98	0.98	5.67E-01
Chr21 genes	ATP5J	Chr21:27096792-27107965	43.08	55.29	1.28	1.46E-05
Chr21 genes	GABPA	Chr21:27113884-27144770	3.66	3.72	1.02	1.00
Chr21 genes	APP	Chr21:27252862-27543138	310.50	373.79	1.20	6.45E-57
Chr21 genes	CYYR1	Chr21:27838529-27945581	5.24	3.99	0.76	3.70E-04
Chr21 genes	ADAMTS1	Chr21:28208608-28217728	94.98	140.94	1.48	7.35E-160
Chr21 genes	ADAMTS5	Chr21:28290232-28339439	6.15	7.14	1.16	2.08E-02
Chr21 genes	NCRNA00113	Chr21:29094698-29123552	<0.5	<0.5	1.00	1.00
Chr21 genes	C21orf94	Chr21:29385682-29395528	<0.5	<0.5	1.00	1.00
Chr21 genes	NCRNA00161	Chr21:29911640-29912676	<0.5	<0.5	1.00	1.00
Chr21 genes	N6AMT1	Chr21:30244513-30257693	2.60	3.35	1.29	3.46E-02

Chr21 genes	LTN1	Chr21:30300466-30365277	3.71	3.21	0.87	2.94E-03
Chr21 genes	RWDD2B	Chr21:30378082-30391685	6.82	9.33	1.37	4.36E-03
Chr21 genes	USP16	Chr21:30396938-30426805	15.51	19.73	1.27	2.36E-05
Chr21 genes	CCT8	Chr21:30428650-30446010	38.64	54.96	1.42	4.92E-20
Chr21 genes	C21orf7	Chr21:30452873-30548200	0.74	1.19	1.62	1.21E-01
Chr21 genes	NCRNA00189	Chr21:30565801-30660525	<0.5	0.70	1.40	7.72E-01
Chr21 genes	BACH1	Chr21:30671220-30734217	6.75	12.90	1.91	2.52E-55
Chr21 genes	GRIK1	Chr21:30909256-31312282	<0.5	<0.5	1.00	1.00
Chr21 genes	NCRNA00258	Chr21:30968360-31003067	0.94	2.61	2.79	4.07E-04
Chr21 genes	GRIK1AS	Chr21:31120494-31136323	<0.5	<0.5	1.00	8.23E-01
Chr21 genes	CLDN17	Chr21:31538241-31538971	<0.5	<0.5	1.00	1.00
Chr21 genes	CLDN8	Chr21:31586325-31588469	<0.5	0.67	1.34	2.70E-02
Chr21 genes	KRTAP24-1	Chr21:31653629-31655276	<0.5	<0.5	1.00	1.00
Chr21 genes	KRTAP25-1	Chr21:31661463-31661832	<0.5	<0.5	1.00	1.00
Chr21 genes	KRTAP26-1	Chr21:31691452-31692607	<0.5	3.52	7.04	2.14E-14
Chr21 genes	KRTAP27-1	Chr21:31709331-31710012	<0.5	<0.5	1.00	1.00
Chr21 genes	KRTAP23-1	Chr21:31720717-31720924	<0.5	<0.5	1.00	1.00
Chr21 genes	KRTAP13-2	Chr21:31743710-31744557	<0.5	<0.5	1.00	1.00
Chr21 genes	MIR4327	Chr21:31747612-31747696	<0.5	<0.5	1.00	1.00
Chr21 genes	KRTAP13-1	Chr21:31768392-31769136	<0.5	<0.5	1.00	1.00
Chr21 genes	KRTAP13-3	Chr21:31797711-31798230	<0.5	<0.5	1.00	1.00
Chr21 genes	KRTAP13-4	Chr21:31802594-31803074	<0.5	<0.5	1.00	1.00
Chr21 genes	KRTAP15-1	Chr21:31812646-31813097	<0.5	<0.5	1.00	1.00
Chr21 genes	KRTAP19-1	Chr21:31852366-31852636	<0.5	0.65	1.30	2.66E-01
Chr21 genes	KRTAP19-2	Chr21:31859511-31859667	<0.5	<0.5	1.00	1.00
Chr21 genes	KRTAP19-3	Chr21:31863782-31864275	<0.5	<0.5	1.00	1.00
Chr21 genes	KRTAP19-4	Chr21:31869175-31869428	<0.5	<0.5	1.00	1.00
Chr21 genes	KRTAP19-5	Chr21:31874191-31874408	<0.5	<0.5	1.00	1.00
Chr21 genes	KRTAP19-6	Chr21:31913854-31914181	<0.5	<0.5	1.00	1.00
Chr21 genes	KRTAP19-7	Chr21:31933418-31933608	<0.5	<0.5	1.00	1.00
Chr21 genes	KRTAP22-2	Chr21:31962425-31962716	<0.5	<0.5	1.00	1.00
Chr21 genes	KRTAP6-3	Chr21:31964759-31965373	<0.5	<0.5	1.00	1.00
Chr21 genes	KRTAP6-2	Chr21:31971006-31971193	<0.5	<0.5	1.00	1.00
Chr21 genes	KRTAP22-1	Chr21:31973440-31973585	<0.5	<0.5	1.00	1.00
Chr21 genes	KRTAP6-1	Chr21:31986006-31986223	<0.5	<0.5	1.00	1.00
Chr21 genes	KRTAP20-1	Chr21:31988774-31988943	<0.5	<0.5	1.00	1.00
Chr21 genes	KRTAP20-4	Chr21:31992946-31993169	<0.5	<0.5	1.00	1.00
Chr21 genes	KRTAP20-2	Chr21:32007583-32007779	<0.5	<0.5	1.00	1.00
Chr21 genes	KRTAP20-3	Chr21:32015183-32015455	<0.5	<0.5	1.00	1.00
Chr21 genes	KRTAP21-3	Chr21:32090843-32091095	<0.5	<0.5	1.00	1.00
Chr21 genes	KRTAP21-2	Chr21:32119271-32119520	<0.5	<0.5	1.00	1.00
Chr21 genes	KRTAP21-1	Chr21:32127459-32127696	<0.5	<0.5	1.00	1.00
Chr21 genes	KRTAP8-1	Chr21:32185016-32185570	<0.5	<0.5	1.00	1.00

Chr21 genes	KRTAP7-1	Chr21:32201360-32202051	<0.5	<0.5	1.00	1.00
Chr21 genes	KRTAP11-1	Chr21:32252966-32253874	<0.5	<0.5	1.00	1.00
Chr21 genes	KRTAP19-8	Chr21:32410479-32410795	<0.5	<0.5	1.00	1.00
Chr21 genes	TIAM1	Chr21:32490736-32931290	2.64	3.79	1.44	2.38E-06
Chr21 genes	SOD1	Chr21:33031935-33041241	135.28	274.15	2.03	2.86E-193
Chr21 genes	SFRS15	Chr21:33043313-33104431	14.38	18.68	1.30	2.42E-08
Chr21 genes	HUNK	Chr21:33245628-33376376	3.53	7.51	2.13	1.04E-45
Chr21 genes	NCRNA00159	Chr21:33452629-33528816	<0.5	<0.5	1.00	1.00
Chr21 genes	C21orf45	Chr21:33640532-33651376	5.68	5.90	1.04	1.00
Chr21 genes	MRAP	Chr21:33664124-33679050	0.79	1.47	1.87	4.98E-01
Chr21 genes	URB1	Chr21:33683330-33765312	3.46	4.47	1.29	7.13E-05
Chr21 genes	SNORA80	Chr21:33749497-33749631	<0.5	<0.5	1.00	1.00
Chr21 genes	C21orf119	Chr21:33765442-33766264	1.02	1.88	1.84	1.62E-01
Chr21 genes	C21orf63	Chr21:33784752-33887697	1.47	2.14	1.46	1.06E-01
Chr21 genes	TCP10L	Chr21:33947152-33957845	<0.5	<0.5	1.00	1.00
Chr21 genes	C21orf59	Chr21:33973984-33984913	20.65	31.26	1.51	2.06E-12
Chr21 genes	SYNJ1	Chr21:34001069-34100351	7.99	8.84	1.11	4.47E-01
Chr21 genes	GCFC1	Chr21:34106213-34144169	7.80	7.89	1.01	8.25E-01
Chr21 genes	C21orf49	Chr21:34144411-34170014	<0.5	<0.5	1.00	5.03E-01
Chr21 genes	C21orf62	Chr21:34162985-34186053	<0.5	<0.5	1.00	1.00
Chr21 genes	OLIG2	Chr21:34398239-34401500	<0.5	<0.5	1.00	1.00
Chr21 genes	OLIG1	Chr21:34442450-34444727	<0.5	<0.5	1.00	1.00
Chr21 genes	C21orf54	Chr21:34537777-34542541	<0.5	<0.5	1.00	1.00
Chr21 genes	IFNAR2	Chr21:34602231-34636818	6.87	7.82	1.14	3.59E-01
Chr21 genes	IL10RB	Chr21:34638672-34669520	42.59	53.84	1.26	1.30E-08
Chr21 genes	IFNAR1	Chr21:34697214-34732126	7.95	8.97	1.13	1.93E-01
Chr21 genes	IFNGR2	Chr21:34775202-34809827	20.64	36.62	1.77	3.81E-41
Chr21 genes	TMEM50B	Chr21:34821449-34852281	8.66	9.50	1.10	8.67E-01
Chr21 genes	DNAJC28	Chr21:34860239-34864023	0.53	0.85	1.60	2.64E-01
Chr21 genes	GART	Chr21:34876239-34915198	11.63	13.94	1.20	3.33E-03
Chr21 genes	SON	Chr21:34915350-34949812	21.24	24.64	1.16	8.36E-06
Chr21 genes	DONSON	Chr21:34950212-34961014	5.21	6.62	1.27	6.64E-02
Chr21 genes	CRYZL1	Chr21:34961650-35014160	9.71	12.03	1.24	4.70E-02
Chr21 genes	ITSN1	Chr21:35014784-35261609	7.51	9.33	1.24	2.20E-05
Chr21 genes	ATP5O	Chr21:35275758-35288158	66.23	90.08	1.36	5.66E-11
Chr21 genes	MRPS6	Chr21:35445823-35515334	45.76	57.01	1.25	8.83E-04
Chr21 genes	SLC5A3	Chr21:35467162-35478560	3.79	2.86	0.75	1.80E-10
Chr21 genes	C21orf82	Chr21:35552978-35562220	<0.5	<0.5	1.00	1.00
Chr21 genes	KCNE2	Chr21:35736323-35743440	<0.5	<0.5	1.00	1.00
Chr21 genes	FAM165B	Chr21:35747749-35761442	3.41	4.49	1.31	3.94E-01
Chr21 genes	KCNE1	Chr21:35818989-35883613	<0.5	<0.5	1.00	4.13E-01
Chr21 genes	RCAN1	Chr21:35888786-35987382	11.87	31.28	2.63	8.32E-114
Chr21 genes	CLIC6	Chr21:36041688-36090519	<0.5	<0.5	1.00	1.00

Chr21 genes	NCRNA00160	Chr21:36096105-36109479	<0.5	<0.5	1.00	1.00
Chr21 genes	RUNX1	Chr21:36160099-36421595	3.19	5.15	1.62	7.13E-16
Chr21 genes	C21orf96	Chr21:36410235-36411723	<0.5	<0.5	1.00	1.00
Chr21 genes	MIR802	Chr21:37093013-37093106	<0.5	<0.5	1.00	1.00
Chr21 genes	SETD4	Chr21:37406840-37432816	4.58	5.90	1.29	1.81E-02
Chr21 genes	CBR1	Chr21:37442285-37445462	17.60	22.57	1.28	3.09E-03
Chr21 genes	CBR3	Chr21:37507263-37518858	4.10	7.75	1.89	7.88E-06
Chr21 genes	DOPEY2	Chr21:37536839-37666571	1.95	2.67	1.37	9.64E-04
Chr21 genes	MORC3	Chr21:37692487-37748943	7.03	7.16	1.02	9.70E-01
Chr21 genes	CHAF1B	Chr21:37757689-37789124	3.25	3.43	1.06	1.00
Chr21 genes	CLDN14	Chr21:37832920-37948867	<0.5	0.68	1.35	6.54E-04
Chr21 genes	SIM2	Chr21:38071991-38122510	<0.5	<0.5	1.00	1.00
Chr21 genes	HLCS	Chr21:38123190-38362536	4.84	5.74	1.18	7.27E-02
Chr21 genes	DSCR6	Chr21:38378863-38391956	<0.5	<0.5	1.00	9.95E-01
Chr21 genes	PIGP	Chr21:38437664-38445458	4.17	5.21	1.25	4.92E-01
Chr21 genes	TTC3	Chr21:38445571-38575406	10.72	10.71	1.00	2.20E-01
Chr21 genes	DSCR9	Chr21:38580955-38592892	<0.5	<0.5	1.00	1.00
Chr21 genes	DSCR3	Chr21:38595726-38639833	9.01	12.34	1.37	1.14E-06
Chr21 genes	DYRK1A	Chr21:38739859-38887678	9.19	12.62	1.37	6.54E-13
Chr21 genes	KCNJ6	Chr21:38996786-39288696	<0.5	<0.5	1.00	3.62E-01
Chr21 genes	DSCR4	Chr21:39426314-39493454	1.66	2.99	1.80	2.18E-02
Chr21 genes	DSCR8	Chr21:39493545-39528604	7.43	13.71	1.84	3.13E-05
Chr21 genes	DSCR10	Chr21:39578250-39580736	<0.5	<0.5	1.00	1.00
Chr21 genes	KCNJ15	Chr21:39628664-39673744	<0.5	<0.5	1.00	7.98E-01
Chr21 genes	ERG	Chr21:39751952-40033704	4.69	5.02	1.07	1.00
Chr21 genes	NCRNA00114	Chr21:40110879-40145401	<0.5	<0.5	1.00	1.00
Chr21 genes	ETS2	Chr21:40177849-40196876	18.13	35.51	1.96	9.44E-89
Chr21 genes	PSMG1	Chr21:40547390-40555440	18.01	23.59	1.31	1.36E-03
Chr21 genes	BRWD1	Chr21:40557404-40685556	4.66	5.88	1.26	1.31E-07
Chr21 genes	NCRNA00257	Chr21:40685861-40686888	0.53	1.46	2.76	1.12E-02
Chr21 genes	HMGN1	Chr21:40714247-40721047	46.55	52.84	1.14	8.24E-02
Chr21 genes	WRB	Chr21:40752213-40769814	7.66	7.49	0.98	7.62E-01
Chr21 genes	LCA5L	Chr21:40777772-40816128	<0.5	<0.5	1.00	1.00
Chr21 genes	SH3BGR	Chr21:40817797-40887426	1.12	1.82	1.62	1.41E-01
Chr21 genes	C21orf88	Chr21:40969077-40984749	<0.5	<0.5	1.00	1.00
Chr21 genes	B3GALT5	Chr21:41029254-41034815	<0.5	<0.5	1.00	1.00
Chr21 genes	IGSF5	Chr21:41117334-41174021	3.59	8.62	2.40	5.29E-19
Chr21 genes	PCP4	Chr21:41239347-41301320	1.92	3.95	2.06	2.44E-02
Chr21 genes	DSCAM	Chr21:41384343-42219039	<0.5	<0.5	1.00	1.37E-03
Chr21 genes	C21orf130	Chr21:42513427-42519991	<0.5	<0.5	1.00	5.38E-01
Chr21 genes	MIR3197	Chr21:42539484-42539556	<0.5	0.57	1.14	1.00
Chr21 genes	BACE2	Chr21:42539728-42648523	21.97	37.16	1.69	2.89E-46
Chr21 genes	PLAC4	Chr21:42547159-42557166	199.51	524.96	2.63	6.32E-321

Chr21 genes	FAM3B	Chr21:42688661-42729654	38.79	29.75	0.77	1.11E-11
Chr21 genes	MX2	Chr21:42733950-42780869	0.85	2.64	3.10	4.66E-13
Chr21 genes	MX1	Chr21:42792520-42831140	5.43	9.25	1.70	1.37E-15
Chr21 genes	TMPRSS2	Chr21:42836479-42880085	0.68	2.44	3.58	4.06E-15
Chr21 genes	NCRNA00111	Chr21:43099462-43117496	<0.5	<0.5	1.00	1.00
Chr21 genes	C21orf129	Chr21:43131680-43135935	<0.5	<0.5	1.00	1.00
Chr21 genes	NCRNA00112	Chr21:43136596-43137741	<0.5	<0.5	1.00	1.00
Chr21 genes	RIPK4	Chr21:43159529-43187249	3.06	5.25	1.71	1.03E-09
Chr21 genes	PRDM15	Chr21:43218387-43299591	1.54	1.59	1.03	1.00
Chr21 genes	C2CD2	Chr21:43305220-43373999	6.05	7.13	1.18	3.94E-02
Chr21 genes	ZNF295	Chr21:43406941-43430496	2.83	3.81	1.35	5.30E-04
Chr21 genes	C21orf121	Chr21:43442113-43445060	<0.5	<0.5	1.00	1.00
Chr21 genes	UMODL1	Chr21:43491426-43563105	<0.5	<0.5	1.00	1.00
Chr21 genes	C21orf128	Chr21:43522244-43528644	<0.5	<0.5	1.00	1.00
Chr21 genes	ABCG1	Chr21:43619799-43717352	2.53	7.58	3.00	6.95E-41
Chr21 genes	TFF3	Chr21:43731778-43735706	<0.5	<0.5	1.00	1.00
Chr21 genes	TFF2	Chr21:43766469-43771208	<0.5	<0.5	1.00	1.00
Chr21 genes	TFF1	Chr21:43782391-43786644	<0.5	<0.5	1.00	1.00
Chr21 genes	TMPRSS3	Chr21:43791996-43816200	<0.5	<0.5	1.00	3.54E-01
Chr21 genes	UBASH3A	Chr21:43824019-43867772	<0.5	<0.5	1.00	1.00
Chr21 genes	RSPH1	Chr21:43892599-43916401	<0.5	<0.5	1.00	1.36E-01
Chr21 genes	SLC37A1	Chr21:43919742-44001549	6.40	13.46	2.10	2.21E-33
Chr21 genes	PDE9A	Chr21:44073862-44195616	4.28	5.11	1.20	3.57E-01
Chr21 genes	WDR4	Chr21:44263206-44299678	4.78	5.66	1.18	3.62E-01
Chr21 genes	NDUFV3	Chr21:44313378-44329772	13.60	21.12	1.55	1.26E-13
Chr21 genes	PKNOX1	Chr21:44394643-44453688	6.22	7.92	1.27	6.58E-04
Chr21 genes	CBS	Chr21:44473301-44496472	5.03	6.08	1.21	1.87E-01
Chr21 genes	U2AF1	Chr21:44513066-44527688	86.54	125.44	1.45	2.07E-27
Chr21 genes	CRYAA	Chr21:44589141-44592913	<0.5	<0.5	1.00	8.23E-01
Chr21 genes	SIK1	Chr21:44834398-44847002	14.67	22.49	1.53	2.21E-30
Chr21 genes	C21orf125	Chr21:44869904-44873769	0.85	0.85	1.00	1.00
Chr21 genes	C21orf84	Chr21:44881974-44898103	0.81	0.80	0.99	1.00
Chr21 genes	HSF2BP	Chr21:44949073-45079374	1.17	1.69	1.45	2.17E-01
Chr21 genes	RRP1B	Chr21:45079432-45115958	5.95	6.00	1.01	8.37E-01
Chr21 genes	PDXK	Chr21:45138978-45182187	18.36	38.85	2.12	2.45E-231
Chr21 genes	CSTB	Chr21:45193832-45196259	60.90	97.71	1.60	1.48E-22
Chr21 genes	RRP1	Chr21:45209418-45223983	12.96	20.38	1.57	3.76E-12
Chr21 genes	LOC284837	Chr21:45225641-45232448	<0.5	<0.5	1.00	2.00E-01
Chr21 genes	AGPAT3	Chr21:45285116-45407474	13.16	14.65	1.11	1.32E-01
Chr21 genes	TRAPPC10	Chr21:45432206-45526430	7.91	10.78	1.36	5.72E-11
Chr21 genes	PWP2	Chr21:45527208-45551062	8.79	17.13	1.95	1.28E-37
Chr21 genes	C21orf33	Chr21:45553494-45565604	38.77	60.73	1.57	1.10E-31
Chr21 genes	ICOSLG	Chr21:45646724-45660834	2.54	6.55	2.58	5.02E-26

Chr21 genes	DNMT3L	Chr21:45666223-45682099	<0.5	<0.5	1.00	9.70E-01
Chr21 genes	AIRE	Chr21:45705763-45718100	<0.5	<0.5	1.00	1.00
Chr21 genes	PFKL	Chr21:45719925-45747260	67.89	118.59	1.75	3.72E-191
Chr21 genes	C21orf2	Chr21:45748827-45759285	7.82	14.39	1.84	1.47E-18
Chr21 genes	TRPM2	Chr21:45773484-45862964	2.41	2.66	1.10	8.97E-01
Chr21 genes	LRRC3	Chr21:45875393-45878730	1.76	1.50	0.85	4.05E-01
Chr21 genes	C21orf29	Chr21:45917775-46131495	<0.5	<0.5	1.00	1.00
Chr21 genes	C21orf90	Chr21:45937098-45938859	<0.5	<0.5	1.00	4.58E-01
Chr21 genes	KRTAP10-1	Chr21:45959068-45960078	<0.5	<0.5	1.00	1.00
Chr21 genes	KRTAP10-2	Chr21:45970318-45971388	<0.5	<0.5	1.00	1.00
Chr21 genes	KRTAP10-3	Chr21:45977906-45978643	<0.5	<0.5	1.00	1.00
Chr21 genes	KRTAP10-4	Chr21:45993606-45994987	<0.5	<0.5	1.00	1.00
Chr21 genes	KRTAP10-5	Chr21:45999564-46000481	<0.5	<0.5	1.00	1.00
Chr21 genes	KRTAP10-6	Chr21:46011150-46012386	<0.5	<0.5	1.00	1.00
Chr21 genes	KRTAP10-7	Chr21:46020497-46022083	<0.5	<0.5	1.00	1.00
Chr21 genes	KRTAP10-8	Chr21:46031996-46032871	<0.5	<0.5	1.00	1.00
Chr21 genes	KRTAP10-9	Chr21:46047040-46048294	<0.5	<0.5	1.00	1.00
Chr21 genes	KRTAP10-10	Chr21:46057273-46058370	<0.5	<0.5	1.00	1.00
Chr21 genes	KRTAP10-11	Chr21:46066331-46067564	<0.5	<0.5	1.00	1.00
Chr21 genes	KRTAP12-4	Chr21:46074130-46074576	<0.5	<0.5	1.00	1.00
Chr21 genes	KRTAP12-3	Chr21:46077849-46078257	<0.5	<0.5	1.00	1.00
Chr21 genes	KRTAP12-2	Chr21:46086107-46086844	<0.5	<0.5	1.00	1.00
Chr21 genes	KRTAP12-1	Chr21:46101493-46102078	<0.5	<0.5	1.00	1.00
Chr21 genes	KRTAP10-12	Chr21:46117087-46117959	<0.5	<0.5	1.00	1.00
Chr21 genes	UBE2G2	Chr21:46188956-46221738	22.60	36.59	1.62	1.04E-39
Chr21 genes	SUMO3	Chr21:46225532-46238044	46.25	59.24	1.28	8.57E-10
Chr21 genes	PTTG1IP	Chr21:46269513-46293741	117.82	223.29	1.90	3.98E-321
Chr21 genes	ITGB2	Chr21:46305868-46348753	24.69	31.12	1.26	1.04E-07
Chr21 genes	C21orf67	Chr21:46353200-46359828	<0.5	0.66	1.31	4.16E-01
Chr21 genes	C21orf70	Chr21:46359955-46396887	3.13	4.84	1.55	5.08E-02
Chr21 genes	NCRNA00163	Chr21:46409781-46414001	<0.5	<0.5	1.00	8.23E-01
Chr21 genes	NCRNA00162	Chr21:46419129-46424642	<0.5	<0.5	1.00	1.00
Chr21 genes	C21orf122	Chr21:46490873-46493126	<0.5	0.96	1.92	2.19E-02
Chr21 genes	ADARB1	Chr21:46494493-46646478	3.53	6.63	1.88	2.39E-15
Chr21 genes	POFUT2	Chr21:46683844-46707811	15.18	22.81	1.50	2.79E-18
Chr21 genes	LOC642852	Chr21:46707967-46717268	2.90	2.99	1.03	1.00
Chr21 genes	COL18A1	Chr21:46825097-46933633	16.67	21.51	1.29	1.83E-14
Chr21 genes	NCRNA00175	Chr21:46839631-46844985	<0.5	<0.5	1.00	1.00
Chr21 genes	SLC19A1	Chr21:46934629-46962351	51.03	65.98	1.29	2.37E-18
Chr21 genes	PCBP3	Chr21:47269875-47362367	<0.5	<0.5	1.00	1.00
Chr21 genes	COL6A1	Chr21:47401663-47424963	531.04	549.80	1.04	1.45E-01
Chr21 genes	COL6A2	Chr21:47518033-47552763	553.67	715.77	1.29	9.13E-292
Chr21 genes	FTCD	Chr21:47556177-47575481	0.75	1.99	2.64	5.88E-06

Chr21 genes	C21orf56	Chr21:47581063-47604373	1.99	4.87	2.45	7.10E-13
Chr21 genes	LSS	Chr21:47608361-47648738	33.34	44.87	1.35	2.81E-26
Chr21 genes	MCM3APAS	Chr21:47649158-47671604	0.57	<0.5	0.87	5.41E-01
Chr21 genes	MCM3AP	Chr21:47655048-47705236	18.52	24.36	1.32	1.17E-15
Chr21 genes	C21orf57	Chr21:47706267-47717664	2.27	4.58	2.02	3.43E-04
Chr21 genes	C21orf58	Chr21:47721050-47743785	3.41	2.91	0.85	1.07E-01
Chr21 genes	PCNT	Chr21:47744036-47865681	5.34	5.97	1.12	2.23E-01
Chr21 genes	DIP2A	Chr21:47878862-47989926	8.71	11.91	1.37	4.04E-14
Chr21 genes	S100B	Chr21:48018531-48025035	<0.5	<0.5	1.00	9.70E-01
Chr21 genes	PRMT2	Chr21:48055527-48084862	30.75	49.23	1.60	1.33E-37
Chr21 genes	LOC100132288	Chr21:9907194-9968585	3.84	6.32	1.64	1.45E-04

Category	gene name	Location	Average($Normal_{RPKM}$)	Average(DS_{RPKM})	(DS_{RPKM}/$Normal_{RPKM}$)	Corrected p-value
Deacetylase	SIRT5	Chr6:13574792-13614783	3.15	2.46	0.78	9.87E-04
Deacetylase	SIRT1	Chr10:69644427-69678146	5.04	3.90	0.77	3.45E-05
Deacetylase	SIRT3	Chr11:215109-236362	4.93	4.92	1.00	9.48E-01
Deacetylase	SIRT4	Chr12:120740124-120751044	0.68	0.91	1.33	8.76E-01
Deacetylase	SIRT7	Chr17:79869815-79876058	11.75	13.45	1.14	3.73E-01
Deacetylase	SIRT2	Chr19:39369195-39390502	14.74	14.90	1.01	8.07E-01
Deacetylase	SIRT6	Chr19:4174107-4182596	7.10	7.70	1.08	1.00
Histone Deacetylase	HDAC1	Chr1:32757708-32799224	50.71	41.00	0.81	8.10E-16
Histone Deacetylase	HDAC4	Chr2:239969865-240322643	1.82	1.62	0.89	1.04E-01
Histone Deacetylase	HDAC11	Chr3:13521715-13547923	6.04	5.70	0.94	3.35E-01
Histone Deacetylase	HDAC3	Chr5:141000443-141016423	24.70	25.21	1.02	8.62E-01
Histone Deacetylase	HDAC2	Chr6:114257322-114292359	17.90	15.01	0.84	8.83E-14
Histone Deacetylase	HDAC9	Chr7:18535369-19036984	0.52	<0.5	0.97	1.09E-01
Histone Deacetylase	HDAC7	Chr12:48176536-48213763	21.69	22.11	1.02	6.86E-01
Histone Deacetylase	HDAC5	Chr17:42154121-42201014	17.19	27.27	1.59	6.50E-47
Histone Deacetylase	HDAC10	Chr22:50683615-50689834	9.95	11.07	1.11	5.69E-01
Histone Deacetylase	HDAC8	Chrx:71549367-71792953	4.83	4.62	0.96	4.46E-01
Histone Deacetylase	HDAC6	Chrx:48660487-48683380	14.81	14.60	0.99	2.35E-01
Lysine Acetyltransferases	HAT1	Chr2:172778935-172848599	18.67	14.14	0.76	1.57E-07
Lysine Acetyltransferases	NCOA1	Chr2:24807346-24993568	6.48	4.93	0.76	3.05E-10
Lysine Acetyltransferases	KAT2B	Chr3:20081524-20195894	4.65	2.99	0.64	4.14E-11
Lysine Acetyltransferases	CLOCK	Chr4:56298660-56412997	1.20	0.83	0.69	3.45E-03
Lysine Acetyltransferases	MYST3	Chr8:41786998-41909505	8.65	7.24	0.84	6.71E-10
Lysine Acetyltransferases	ELP3	Chr8:27950584-28048667	8.99	7.86	0.87	7.08E-03
Lysine Acetyltransferases	GTF3C4	Chr9:135545789-135565468	5.12	4.10	0.80	9.31E-04
Lysine Acetyltransferases	MYST4	Chr10:76586379-76792639	4.39	3.79	0.86	4.86E-04
Lysine Acetyltransferases	KAT5	Chr11:65479489-65484453	17.17	22.70	1.32	2.54E-04
Lysine Acetyltransferases	CREBBP	Chr16:3775058-3930121	12.46	10.29	0.83	2.38E-16
Lysine Acetyltransferases	MYST1	Chr16:31128985-31142713	16.24	22.02	1.36	2.89E-05

Lysine Acetyltransferases	MYBBP1A	Chr17:4442734-4458681	7.98	6.30	0.79	4.10E-06
Lysine Acetyltransferases	MYST2	Chr17:47866071-47906456	8.88	7.89	0.89	7.81E-03
Lysine Acetyltransferases	KAT2A	Chr17:40265129-40273382	20.88	20.95	1.00	4.49E-01
Lysine Acetyltransferases	NCOA3	Chr20:46130601-46285620	25.68	24.85	0.97	2.19E-04
Lysine Acetyltransferases	EP300	Chr22:41488614-41576080	22.66	17.85	0.79	2.62E-35
Lysine Acetyltransferases	TAF1	Chrx:70586114-70685854	4.77	4.00	0.84	7.72E-05
Lysine Demethylases	KDM5B	Chr1:202696533-202777549	13.34	11.40	0.85	9.93E-09
Lysine Demethylases	KDM4A	Chr1:44115797-44171188	10.92	9.57	0.88	2.41E-04
Lysine Demethylases	KDM1A	Chr1:23345941-23410184	20.79	18.67	0.90	3.81E-04
Lysine Demethylases	KDM3A	Chr2:86668271-86719838	5.91	8.39	1.42	4.46E-08
Lysine Demethylases	Kdm3b	Chr5:137688285-137772716	13.54	12.92	0.95	9.59E-03
Lysine Demethylases	KDM4C	Chr9:6720863-7175647	3.67	3.61	0.98	6.10E-01
Lysine Demethylases	KDM2A	Chr11:66886740-67025542	23.69	22.09	0.93	1.73E-06
Lysine Demethylases	KDM4D	Chr11:94706845-94732674	<0.5	<0.5	1.00	1.00
Lysine Demethylases	KDM5A	Chr12:389223-498515	5.79	4.83	0.83	5.81E-08
Lysine Demethylases	KDM2B	Chr12:121866902-122018920	5.51	4.77	0.86	2.13E-03
Lysine Demethylases	KDM6B	Chr17:7743235-7758118	12.99	14.90	1.15	1.44E-02
Lysine Demethylases	KDM4B	Chr19:4969124-5153606	15.89	18.35	1.16	5.30E-03
Lysine Demethylases	KDM6A	Chrx:44732423-44971843	4.07	3.91	0.96	3.76E-01
Lysine Demethylases	KDM5C	Chrx:53220504-53254604	23.15	24.92	1.08	4.42E-01
Lysine Demethylases	KDM5D	Chry:21867303-21906825	9.29	12.89	1.39	5.24E-22
Lysine Methyltransferase	PRDM2	Chr1:14026735-14151572	7.71	5.78	0.75	7.77E-18
Lysine Methyltransferase	ASH1L	Chr1:155305053-155491409	6.15	4.66	0.76	2.68E-16
Lysine Methyltransferase	SMYD2	Chr1:214454565-214510477	33.31	30.06	0.90	1.34E-03
Lysine Methyltransferase	SETDB1	Chr1:150898815-150937220	12.56	12.29	0.98	1.92E-01
Lysine Methyltransferase	SETD2	Chr3:47057900-47205467	9.48	7.67	0.81	2.77E-12
Lysine Methyltransferase	SETD7	Chr4:140427193-140477577	12.91	9.82	0.76	1.52E-20
Lysine Methyltransferase	NSD1	Chr5:176560080-176727213	7.05	6.33	0.90	6.17E-06
Lysine Methyltransferase	EHMT2	Chr6:31847537-31865464	<0.5	<0.5	1.00	1.00
Lysine Methyltransferase	EZH2	Chr7:148504475-148581414	14.61	9.20	0.63	1.51E-19
Lysine Methyltransferase	MLL5	Chr7:104654637-104754531	9.69	7.45	0.77	4.26E-14
Lysine Methyltransferase	MLL3	Chr7:151832012-152133090	5.44	5.07	0.93	3.47E-04
Lysine Methyltransferase	EHMT1	Chr9:140513444-140730576	13.33	12.80	0.96	3.83E-02
Lysine Methyltransferase	SUV39H2	Chr10:14920782-14946302	2.29	1.42	0.62	1.28E-04
Lysine Methyltransferase	SUV420H1	Chr11:67923507-67980784	8.09	5.76	0.71	8.11E-15
Lysine Methyltransferase	MLL	Chr11:118307205-118395934	1.83	1.31	0.71	8.77E-09
Lysine Methyltransferase	MLL2	Chr12:49412762-49449107	12.87	8.74	0.68	1.04E-93
Lysine Methyltransferase	SETD1B	Chr12:122242638-122270561	10.70	8.81	0.82	7.90E-12
Lysine Methyltransferase	SETD8	Chr12:123868704-123893898	8.82	7.75	0.88	1.65E-02
Lysine Methyltransferase	SETDB2	Chr13:50018429-50069138	1.81	1.11	0.61	8.57E-07
Lysine Methyltransferase	SETD1A	Chr16:30968615-30995981	7.25	7.33	1.01	7.39E-01
Lysine Methyltransferase	SUV420H2	Chr19:55851221-55859488	8.58	7.47	0.87	2.73E-02
Lysine Methyltransferase	MLL4	Chr19:36208921-36229778	12.43	11.43	0.92	4.88E-05

Lysine Methyltransferase	DOT1L	Chr19:2164148-2232576	12.68	15.36	1.21	6.59E-06
Lysine Methyltransferase	SUV39H1	Chrx:48555131-48567404	5.96	5.88	0.99	7.71E-01
	DNMT1	Chr19:10244023-10305755	74.80	66.32	0.89	1.72E-25
	DNMT3A	Chr2:25455846-25565459	25.11	23.89	0.95	2.67E-04
	DNMT3B	Chr20:31350191-31397161	4.93	3.82	0.77	4.58E-05
	DNMT3L	Chr21:45666223-45682099	<0.5	<0.5	1.00	9.70E-01
	TET1	Chr10:70320117-70454238	0.80	0.57	0.71	5.89E-03
	TET2	Chr4:106067943-106200958	4.01	2.54	0.63	5.18E-19
	TET3	Chr2:74273450-74335300	5.44	3.40	0.62	1.14E-29
	MBD1	Chr18:47795217-47808144	19.27	20.30	1.05	1.00
	MBD2	Chr18:51680575-51751158	16.83	20.02	1.19	7.25E-03
	MBD3	Chr19:1576678-1592652	47.67	68.57	1.44	1.17E-35
	MBD4	Chr3:129149793-129158852	9.96	7.78	0.78	2.48E-05
	MECP2	Chrx:153287264-153363188	4.93	3.81	0.77	7.25E-11
	MBD3L1	Chr19:8953269-8954016	<0.5	<0.5	1.00	1.00
	NFAT5	Chr16:69598997-69738553	9.72	7.25	0.75	8.44E-34
	RCAN2	Chr6:46188469-46293531	<0.5	<0.5	1.00	1.00
	RCAN3	Chr1:24829387-24862425	2.48	2.15	0.87	4.40E-01
	COL6A4P1	Chr3:15206869-15247466	<0.5	<0.5	1.00	1.00
	COL6A4P2	Chr3:129931663-129992648	<0.5	<0.5	1.00	1.00
	COL6A5	Chr3:130064359-130203688	<0.5	<0.5	1.00	7.32E-01
	COL6A6	Chr3:130279178-130395887	5.02	2.77	0.55	1.09E-31
	REST	Chr4:57774042-57802009	5.73	3.41	0.60	1.30E-25
