# Supplementary material for: Global DNA Hypermethylation in Down Syndrome Placenta
Source: PLoS Genet. 2013 Jun 6;9(6):e1003515. doi: 10.1371/journal.pgen.1003515 (PMC3675012; doi:10.1371/journal.pgen.1003515)
Supplement: Table S5 — Differentiated methylated genes with causal association to DS. (DOCX) [file pgen.1003515.s014.docx]

**Supplemental Table 5** Differentiated methylated genes with causal association to DS.

| **Gene** | **Description** | **Link to DS** | **Average promoter methylation in DS** | **Average promoter methylation in normal samples** |
| --- | --- | --- | --- | --- |
| COL6A1 | collagen 6 A1 | RNA Overexpression in DS fetuses compared to euploid | 71.5 | 60.6 |
| DPYSL4 | dihydropyrimidinase-like 4 | Protein overexpression in DS brains | 27.8 | 14.3 |
| DSCAM | Down syndrome cell adhesion molecule | maps to a DS critical region of chromosome 21q22.2-22.3 | 51.2 | 32.3 |
| DSCR6 | Down Syndrome critical region gene 6 | maps to a DS critical region of chromosome 21q22.2-22.3 | 54.7 (promoter1)  37.8 (promoter2) | 39.5 (promoter1)  24.4 (promoter2) |
| NPAS4 | neuronal PAS domain protein 4 | Directly inhibited by Sim2 a transcriptional repressor important in DS | 33.0 | 18.0 |
| SNAP25 | synaptosomal-associated protein, 25kDa | RNA overexpressed in adult DS brains | 24.2 | 14.2 |
| ZAP70 | zeta-chain (TCR) associated protein kinase 70kDa | Decreased tyrosine phosphoylation in DS children lymphocytes | 69.5 | 51.8 |
| LEP | Leptin | Protein levels decreased in DS amniotic fluid | 48.7 | 37.5 |
