# Supplementary material for: Global DNA Hypermethylation in Down Syndrome Placenta
Source: PLoS Genet. 2013 Jun 6;9(6):e1003515. doi: 10.1371/journal.pgen.1003515 (PMC3675012; doi:10.1371/journal.pgen.1003515)
Supplement: Table S6 — Top 10 transcription factors whose target genes were either enriched or depleted for differentially methylated promoters in DS. (DOCX) [file pgen.1003515.s015.docx]

**Supplemental Table 6** Top 10 transcription factors whose target genes were either enriched or depleted for differentially methylated promoters in DS.

| **Network Object Name** | **Actual** | **n** | **R** | **N** | **Expected** | **Ratio** | **p-value** | **z-score** |
| --- | --- | --- | --- | --- | --- | --- | --- | --- |
| \| HNF4-alpha \| \| --- \| | 41 | 559 | 1962 | 13799 | 79.48 | 0.5158 | 2.01E-07 | -4.758 |
| NRSF | 32 | 559 | 307 | 13799 | 12.44 | 2.573 | 9.39E-07 | 5.727 |
| CSX(Nkx2.5) | 21 | 559 | 160 | 13799 | 6.482 | 3.24 | 1.97E-06 | 5.856 |
| PBX1 | 13 | 559 | 69 | 13799 | 2.795 | 4.651 | 3.31E-06 | 6.247 |
| HNF3-beta | 25 | 559 | 259 | 13799 | 10.49 | 2.383 | 5.31E-05 | 4.616 |
| VDR | 23 | 559 | 230 | 13799 | 9.317 | 2.469 | 6.10E-05 | 4.615 |
| PAX2 | 8 | 559 | 36 | 13799 | 1.458 | 5.486 | 7.64E-05 | 5.537 |
| SP1 | 127 | 559 | 2331 | 13799 | 94.43 | 1.345 | 0.0001797 | 3.753 |
| Miz-1 | 8 | 559 | 41 | 13799 | 1.661 | 4.817 | 0.0002019 | 5.029 |
| AP-2A | 38 | 559 | 517 | 13799 | 20.94 | 1.814 | 0.0002859 | 3.878 |

***Actual*** number of targets in the activated dataset(s) regulated by the chosen transcription

factor (TF)

***n*** number of network objects in the activated dataset(s)

***R*** number of targets in the complete database or background list regulated by the chosen

TF

***N*** total number of gene-based objects in the complete database or background list

***Expected*** mean value for hypergeometric distribution (n*R/N)

***Ratio*** connectivity ratio (Actual/Expected)

***z-score*** z-score ((Actual-Expected)/sqrt(variance))

***p-value*** probability to have the given value of Actual or higher (or lower for negative z-score)
