# Supplementary material for: Global DNA Hypermethylation in Down Syndrome Placenta
Source: PLoS Genet. 2013 Jun 6;9(6):e1003515. doi: 10.1371/journal.pgen.1003515 (PMC3675012; doi:10.1371/journal.pgen.1003515)
Supplement: Table S7 — EpiTYPER assays for DNA methylation validation. (DOCX) [file pgen.1003515.s016.docx]

**Supplemental Table 7** EpiTYPER assays for DNA methylation validation.
