# Supplementary material for: Global DNA Hypermethylation in Down Syndrome Placenta
Source: PLoS Genet. 2013 Jun 6;9(6):e1003515. doi: 10.1371/journal.pgen.1003515 (PMC3675012; doi:10.1371/journal.pgen.1003515)
Supplement: Table S8 — Quantitative real-time PCR assays for gene expression validation. (DOCX) [file pgen.1003515.s017.docx]

**Supplemental Table 8** QPCR assays for gene expression validation.

| Gene Name | Forward Primer (5'->3') | Reverse Primer (5'->3') | Amplicon Size (bp) |
| --- | --- | --- | --- |
| TET1 | TGGAAAGTCTCACTTCTGAGGAGAA | GGCCAGCGACCAAAACC | 76 |
| TET2 | GGACTGAGCTGCTGAATTCAACT | CCTCAACATGGTTGGTTCTATCC | 91 |
| TET3 | GGCTTCTTGGAGTCACCTCTTAAG | CCACTATTTGTTCGACGCAATC | 118 |
| REST | AAACCATTTTCCAAGGAAAGTATACAC | TGGGCGTTCTCCTGTATGAGTT | 106 |
| CES1 | TCTTGGACCCCTGAGGTTTACTC | TTGGGTGCACATAGGAGGGTA | 91 |
| TFAP2E | GGACCTGCAGGCAATGGA | GGATGGGCACTTTCTTGATCA | 71 |
| CDH13 | TGCAGTTATGTTTCTCAGAGCAACT | AATTCTAAACTTGACCTTCAGTGACTGT | 121 |
| NDN | ACATCTTTCACCATGTCTGGAAAC | GCGCAGCTGGTGCAGAA | 98 |
| GAPDH | TTCATTGACCTCAACTACATGGTTTAC | TGACAAGCTTCCCGTTCTCA | 97 |
