# Supplementary material for: Global DNA Hypermethylation in Down Syndrome Placenta
Source: PLoS Genet. 2013 Jun 6;9(6):e1003515. doi: 10.1371/journal.pgen.1003515 (PMC3675012; doi:10.1371/journal.pgen.1003515)
Supplement: Supplementary file 10 [file pgen.1003515.s005.docx]

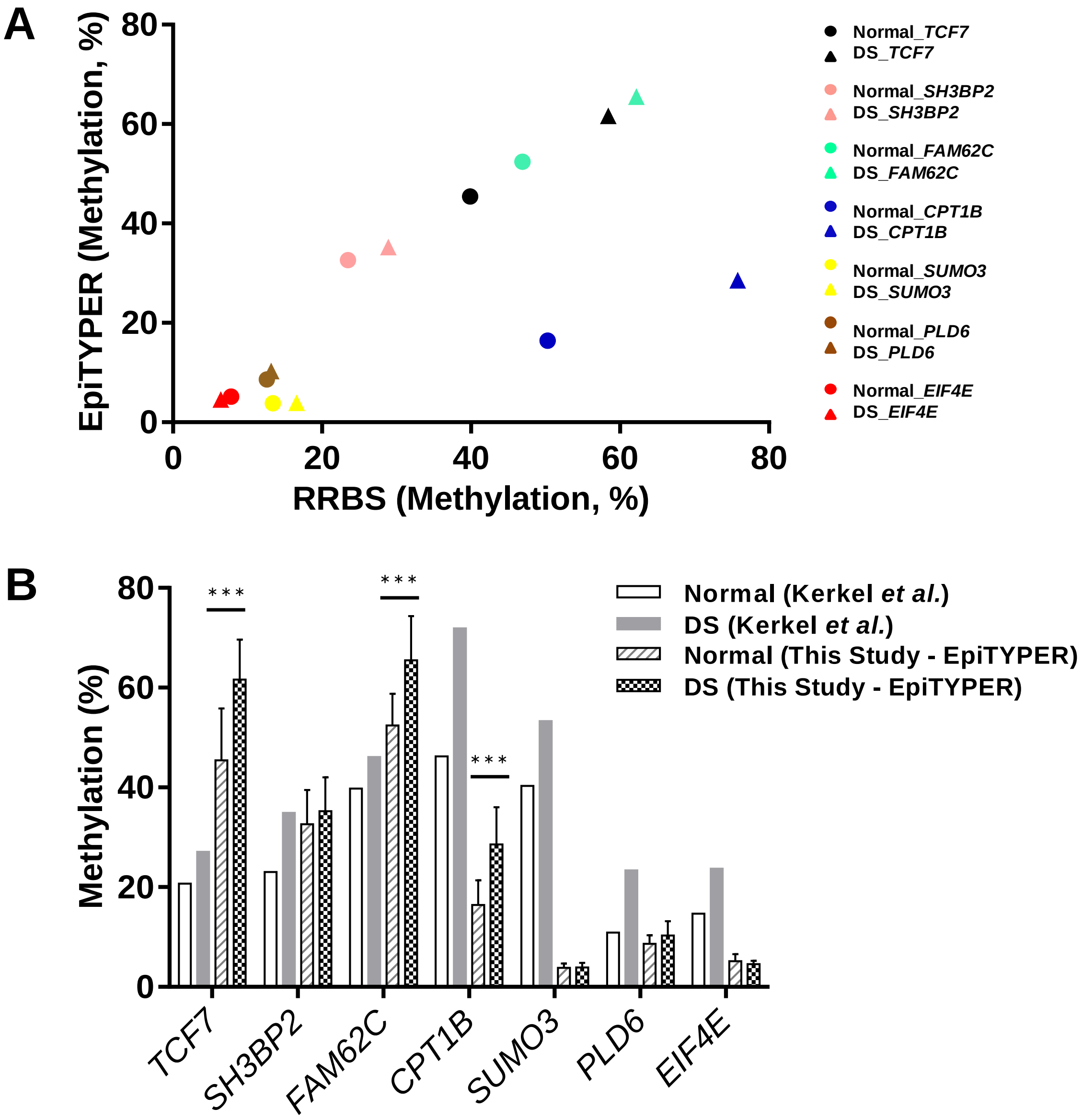
A
EpiTYPER (Methylation, %)
80
60
40
20
0
0
20
40
60
80
RRBS (Methylation, %)
Normal_TCF7
DS_TCF7
Normal_SH3BP2
DS_SH3BP2
Normal_FAM62C
DS_FAM62C
Normal_CPT1B
DS_CPT1B
Normal_SUMO3
DS_SUMO3
Normal_PLD6
DS_PLD6
Normal_EIF4E
DS_EIF4E
B
Methylation (%)
80
60
40
20
0
***
***
***
Normal (Kerkel et al.)
DS (Kerkel et al.)
Normal (This Study - EpiTYPER)
DS (This Study - EpiTYPER)
TCF7
SH3BP2
FAM62C
CPT1B
SUMO3
PLD6
EIF4E
