# Supplementary material for: Global DNA Hypermethylation in Down Syndrome Placenta
Source: PLoS Genet. 2013 Jun 6;9(6):e1003515. doi: 10.1371/journal.pgen.1003515 (PMC3675012; doi:10.1371/journal.pgen.1003515)
Supplement: Supplementary file 11 [file pgen.1003515.s006.docx]

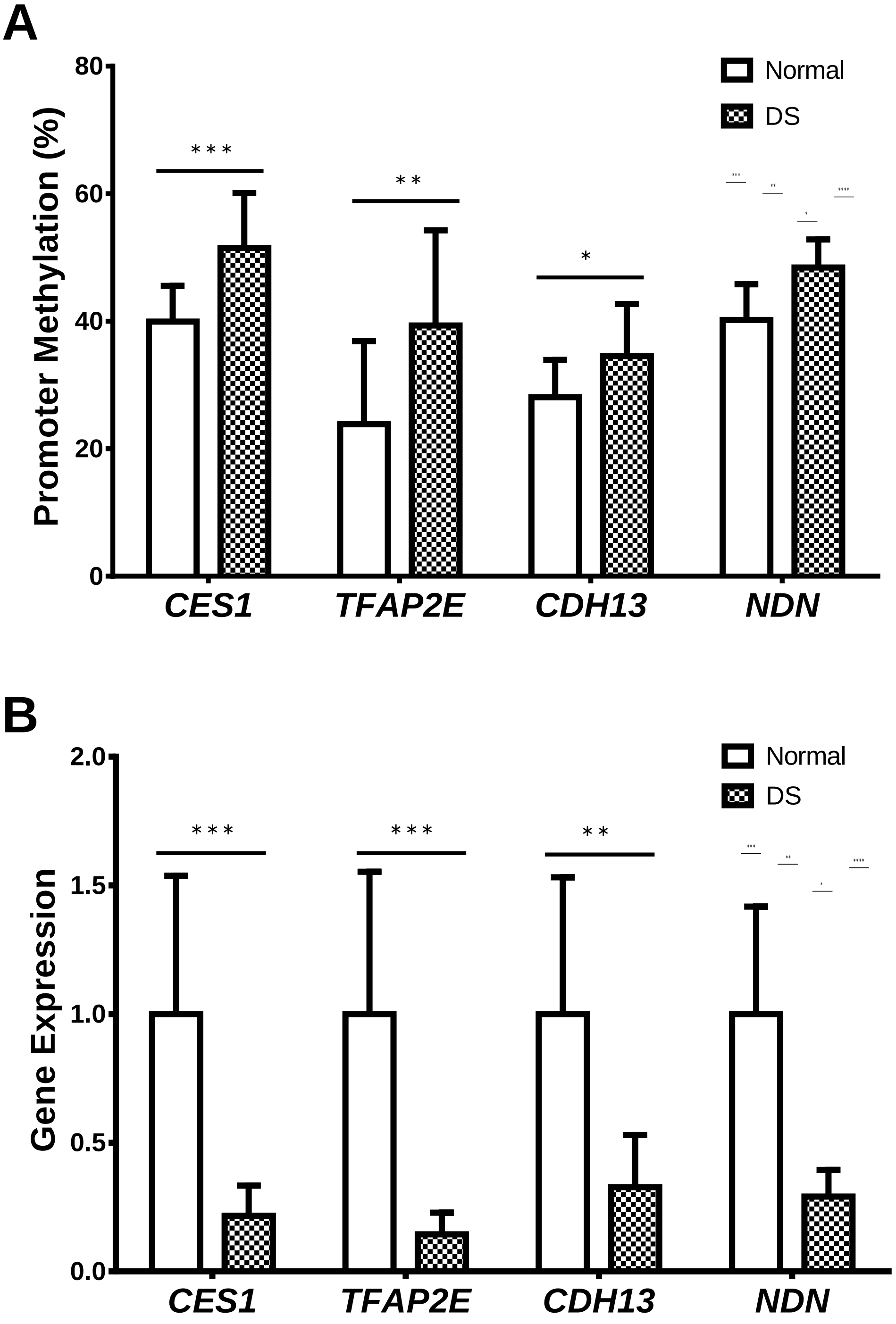
A
Promoter Methylation (%)
80
60
40
20
0
Normal
DS
***
**
*
CES1
TFAP2E
CDH13
NDN
B
Gene Expression
2.0
1.5
1.0
0.5
0.0
Normal
DS
***
***
**
CES1
TFAP2E
CDH13
NDN
