# Supplementary material for: Global DNA Hypermethylation in Down Syndrome Placenta
Source: PLoS Genet. 2013 Jun 6;9(6):e1003515. doi: 10.1371/journal.pgen.1003515 (PMC3675012; doi:10.1371/journal.pgen.1003515)
Supplement: Supplementary file 12 [file pgen.1003515.s007.docx]

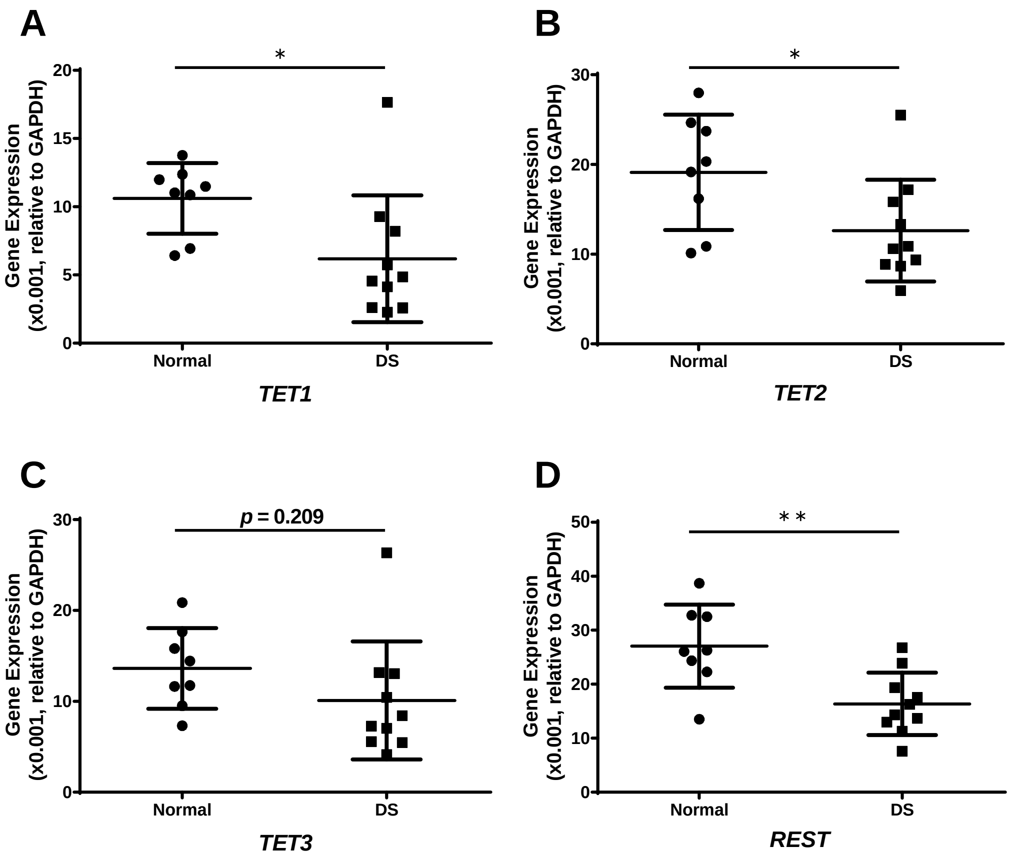
A
B
C
D
Gene Expression
(x0.001, relative to GAPDH)
Normal
DS
TET1
TET2
TET3
REST
*
**
p = 0.209
